# Sweroside: unveiling broad therapeutic potential—from mechanistic insights to clinical potential

**DOI:** 10.3389/fphar.2025.1594278

**Published:** 2025-07-29

**Authors:** Liu Peng, Man Zuo, Tian Qiu, Wenying Lan, Yue Wen, Xiao-Qin Ye

**Affiliations:** ^1^Division of Gastrointestinal Surgery, Department of General Surgery, West China Hospital, Sichuan University, Chengdu, China; ^2^School of Pharmacy, Chengdu University of Traditional Chinese Medicine, Chengdu, China; ^3^Keele Joint Health and Medical Sciences Institute, Chengdu University of Traditional Chinese Medicine, Chengdu, China; ^4^ Traditional Chinese Medicine Hospital of Meishan, Meishan, Sichuan, China

**Keywords:** Sweroside, iridoid glycosides, anti-inflammatory, antioxidant, metabolic disorders, natural product

## Abstract

**Background:**

Sweroside, a secoiridoid glycoside abundantly present in medicinal plants, has garnered significant attention due to its diverse bioactivities, including anti-inflammatory, antioxidant, hepatoprotective, antidiabetic, antibacterial, and anticancer effects. Additionally, it exhibits promising potential in neuroprotection and dermatological health.

**Purpose:**

This review aims to provide a comprehensive analysis of Sweroside, focusing on its sources, biosynthetic pathways, pharmacodynamic mechanisms, and therapeutic potential. The insights presented aim to facilitate the development of novel pharmacotherapies and advance precision medicine.

**Methods:**

A systematic review of the literature was conducted using databases such as PubMed, Web of Science, and Google Scholar. The study examined Sweroside’s sources, distribution, biosynthesis, pharmacodynamics, and therapeutic applications. Challenges in its clinical translation were also evaluated, with proposed strategies to enhance bioavailability.

**Results:**

Sweroside modulates critical signaling pathways, such as NF-κB, mTOR, MAPK, and PI3K/Akt, and their molecular substrates, contributing to its therapeutic effects across various pathological conditions. Preclinical studies demonstrate its efficacy in treating hepatic disorders, osteoporosis, cardiovascular diseases, and neurological dysfunctions. It also shows promise in neuroprotection and dermatological health. However, clinical translation is hindered by its low bioavailability and rapid metabolic degradation.

**Conclusion:**

Sweroside is a versatile natural metabolite with multi-target therapeutic potential, positioning it as a promising candidate for next-generation pharmacotherapies. To realize its clinical potential, future research should prioritize optimizing its pharmacokinetics, including enhancing bioavailability and developing advanced drug delivery systems. Further investigation into its molecular mechanisms and intracellular targets across diverse pathological conditions is essential. Sweroside’s integration into precision medicine offers significant opportunities for addressing chronic and complex diseases.

## Graphical Abstract

**Figure:**
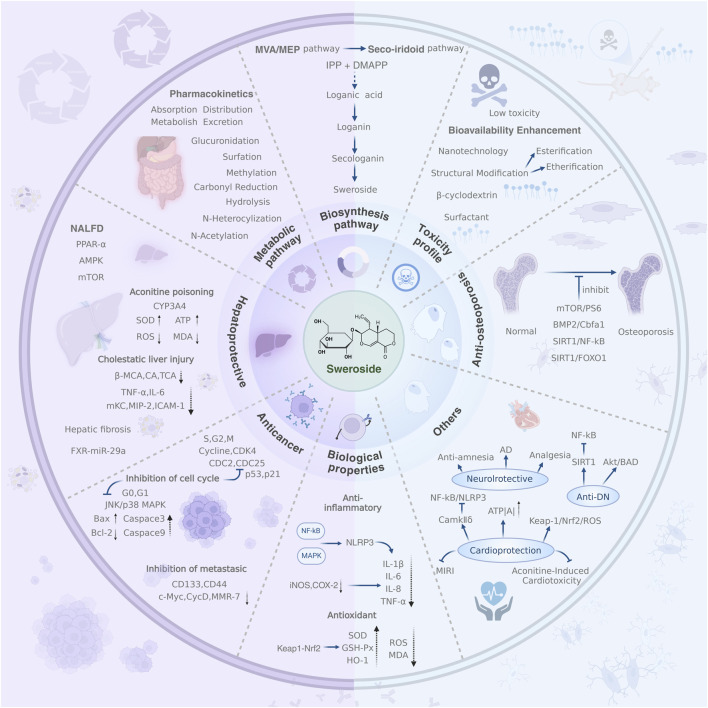
This review provides a comprehensive overview of Sweroside, encompassing its biosynthesis, pharmacokinetics, biological activities, and therapeutic potential. It emphasizes Sweroside’s multi-target effects, including hepatoprotection, anti-inflammatory, and neuroprotective properties, mediated through critical signaling pathways. Additionally, it explores strategies to enhance bioavailability, such as nanotechnology and structural modifications, highlighting its potential in tackling complex diseases.

## 1 Introduction

Natural products, organic compounds derived from organisms in nature, occupy a pivotal role in drug discovery due to their unique biological activities and multifunctional therapeutic potential. In recent years, natural products sourced from medicinal plants have garnered significant attention in scientific research, primarily owing to their exceptional pharmacological properties and relatively low toxicity. Among these, iridoid glycosides (Fadzil et al., 2021) have emerged as a prominent class of bioactive metabolites, with Sweroside standing out as a typical representative.

Sweroside, first isolated from Swertia chirayita, is widely distributed among Gentianaceae plants, particularly in the Qinghai-Tibet Plateau and the source region of the Yellow River (Zhou D. et al., 2021). In traditional medicine, Swertia chirayita—referred to as “Dida” or “Zangyinchen” in Tibet, Yunnan, and Guizhou provinces of China—has long been revered for its potent anti-hepatitis, antipyretic, and detoxifying properties (Wang et al., 2013). Beyond Swertia chirayita, other species within the Swertia genus also exhibit a broad spectrum of pharmacological activities, including anticancer, antidiabetic, antibacterial, antioxidant, and neuroprotective effects. These diverse therapeutic applications underscore the likelihood that Sweroside serves as a key bioactive metabolite underpinning the efficacy of these traditional remedies.

Extensive research has further highlighted Sweroside’s remarkable pharmacological profile, encompassing anti-inflammatory, antioxidant, analgesic, and neuroprotective effects. Notably, it demonstrates significant therapeutic potential in addressing a range of major diseases, including liver disorders, osteoporosis, cancer, rheumatoid arthritis, diabetic nephropathy, myocardial injury, and Alzheimer’s disease (Yang et al., 2022). These multifaceted properties have positioned Sweroside as a focal point in the development of novel therapeutic strategies. However, its successful clinical translation necessitates a deeper understanding of its intricate molecular mechanisms and diverse biological targets. Mechanistically, Sweroside’s antioxidant and anti-inflammatory properties are particularly noteworthy, offering critical insights into its therapeutic potential. Studies have revealed that Sweroside effectively attenuates inflammatory responses by suppressing the release of pro-inflammatory cytokines (e.g., TNF-α, IL-1β, IL-6, and IL-8) through inhibition of the NF-κB/NLRP3 signaling pathway. Furthermore, it promotes osteoblast differentiation by modulating key molecules associated with cellular proliferation and differentiation, such as phosphorylated mammalian target of rapamycin (P-mTOR), phosphorylated S6 protein (PS6), Runt-related transcription factor 2 (RUNX2), and Osterix (OSX). These findings provide robust theoretical support for its potential application in treating bone metabolism disorders. Additionally, Sweroside exerts precise control over apoptosis by regulating apoptosis-related proteins, including Bcl-2 and Bax, further highlighting its multidimensional biological effects. Collectively, these mechanisms establish a solid scientific foundation for the application of Sweroside in combating chronic diseases.

Despite significant advancements in understanding the pharmacological activities and mechanisms of Sweroside, current research remains fragmented and lacks systematic integration. This review seeks to address this gap by providing a comprehensive synthesis of Sweroside’s distribution in plants, biosynthetic pathways, clinical applications, molecular mechanisms, and recent progress in metabolism and toxicology. By consolidating current insights, this review aims to construct a clearer scientific framework for Sweroside as a promising natural therapeutic agent and to lay a robust theoretical foundation for its future academic exploration and clinical development.

## 2 Methods

### 2.1 Search strategy

We comprehensively searched databases including PubMed, Web of Science, Elsevier ScienceDirect, and Google Scholar. The main search terms used were “Sweroside”, “anti-inflammatory”, “biosynthesis”, “toxicology”, and “therapeutic”, which were combined using Boolean operators (AND/OR). The search covered all published articles. The titles and abstracts of the retrieved articles were then reviewed.

### 2.2 Selection criteria

Inclusion criteria: Studies on biosynthetic precursors and gene regulation of Sweroside; Research involving *in vivo* or *in vitro* metabolism of Sweroside; Original studies providing experimental data, with clear experimental methods, reasonable control groups, and reliable data.

Exclusion criteria: Studies irrelevant to Sweroside; Studies without experimental verification; Review articles.

### 2.3 Data analysis

This review comprehensively analyzed data related to the pharmacokinetics, biological activities, therapeutic effects, and toxicity of Sweroside. This included improvements in various diseases by Sweroside in different cell and animal models, its effects on inflammation mediators and oxidative stress markers associated with various diseases, as well as the drug’s metabolic profile.

### 2.4 Search results

A total of 4,450 articles related to Sweroside were found in Google Scholar, 747 in Elsevier ScienceDirect, 273 in Web of Science, and 281 in PubMed. After screening according to the inclusion and exclusion criteria, 31 articles were finally selected.

## 3 The plant sources and related biosynthetic pathways of swroside

Sweroside, a secoiridoid metabolite predominantly derived from plants of the Gentianaceae family, is most abundantly found in species of the genus Swertia. For centuries, Swertia plants have been integral to traditional medicine, celebrated for their heat-clearing and detoxifying properties. Their therapeutic applications are well-documented in various traditional medical systems, including the Indian Pharmacopoeia, British herbal medicine, Ayurveda, Unani, and Siddha (Kumar and Van Staden, 2016). These systems have employed Swertia plants to treat a wide array of ailments, such as malaria, liver diseases, jaundice, cholecystitis, pneumonia, and diabetes (Kshirsagar et al., 2019). In Tibetan and Yunnan regions of China, Swertia plants—referred to as “Dida” or “Zangyinchen”—are cornerstone components of Tibetan traditional medicine, particularly renowned for their efficacy in treating liver and gallbladder disorders. Globally, approximately 170 species of Swertia have been identified, with the majority distributed across Asia and smaller populations found in the Americas and Africa. The Tibetan Plateau in Southwest China serves as a biodiversity hotspot for this genus, hosting approximately 79 species, of which nearly 40 possess confirmed medicinal value (Tong et al., 2015). Similarly, India has recorded around 40 species of Swertia. Beyond the Swertia genus, Sweroside has also been isolated from other medicinal plants, including Lonicera (Kumar et al., 2000; Liang et al., 2013a), *Cornus officinalis* Sieb. et Zucc. (Huang et al., 2018), and *Gentiana macrophylla* Pall. (Liang et al., 2013b). This extensive diversity of plant resources, coupled with their wide geographical distribution, provides a robust foundation for the exploration and application of Sweroside in pharmacological research.

Chemically, Sweroside is an odorless, bitter-tasting powder characterized by its high stability (Jovanovic et al., 2021), making it a promising candidate for applications in the pharmaceutical and food industries. Modern pharmacological studies have revealed its multifaceted bioactivities, including anti-inflammatory, antioxidant, analgesic, hepatoprotective, antidiabetic, and antimicrobial effects. For instance, Sweroside has been shown to exert significant anti-inflammatory effects by downregulating the expression of pro-inflammatory cytokines through inhibition of the NF-κB/NLRP3 signaling pathway. Furthermore, it has demonstrated potential in the treatment of diabetes, neurodegenerative diseases, and certain toxicological conditions (Deng et al., 2013). These discoveries not only validate the traditional medicinal applications of Sweroside but also establish a robust scientific foundation for its exploration in novel therapeutic domains.

In recent years, derivatives of Sweroside have garnered attention for their expanded pharmacological potential, including anti-inflammatory, anticancer, and neuroregenerative properties. For example, a derivative with potent anti-inflammatory effects has been isolated from Gentiana scabra (He et al., 2015). Additionally, chemically synthesized Sweroside derivatives containing diindolylmethane structures have shown efficacy in inhibiting the proliferation of specific cancer cells. Notably, these derivatives, when combined with paclitaxel, significantly reverse drug resistance, highlighting their potential in overcoming therapeutic challenges (Pan, 2023). Collectively, these advancements not only broaden the application spectrum of Sweroside and its derivatives but also underscore their promise as candidates for further development in anti-inflammatory and anticancer therapies.

### 3.1 Sweroside: botanical sources

Sweroside is predominantly found in plants of the Gentianaceae family, particularly within the genus Gentiana, and in species of the Cornaceae family, notably the genus Cornus. These plants have captured considerable scientific interest due to their pronounced hepatoprotective, antitumor, and anti-inflammatory activities, alongside a range of other biological properties. Furthermore, Sweroside’s occurrence in additional medicinal plants has broadened its potential for research and therapeutic applications. Understanding the distribution patterns of Sweroside holds significant importance for advancing the development and sustainable utilization of medicinal plant resources. This review systematically examines its distribution across various plant species (Table 1).

**TABLE 1:** Distribution of sweroside in various plants and their corresponding extraction solvents.

Plant	Part	Reference
*Gentiana rigescens* Franch. ex Hemsl.	Rhizome, leaves, flowers	Zhang et al. (2020)
*Centaurium erythraea* Rafn	Aerial parts	Nikolic et al. (2023)
*Gentiana asclepiadea* L.	Roots	Mihailovic et al. (2013a)
*Gentiana gelida* M.Bieb. , *Gentiana septemfida *Pallas	Herbaceous parts and roots	Olennikov et al. (2019)
*Gentiana macrophylla* Pall.	Roots	Tan et al. (1996)
*Gentiana purpurea* L.	Roots, leaves	Zhang et al. (2023)
*Gentiana lutea* L., *Gentiana boissieri* Schott and Kotschy ex Boiss.	Roots	Asci et al. (2022)
*Gentiana clusii* Perr. and Songeon	Buds	Krstic-Milosevic et al. (2020)
*Gentiana straminea* Maxim.	Roots	Wei et al. (2012)
*Gentiana macrophylla* Pall	Flowers	Jia et al. (2016)
*Gentiana cruciata* L.	Aerial parts, roots	Mihailović et al. (2014)
*Gentiana macrophylla* Pall.	Aerial parts	Wu et al. (2012)
*Gentiana veitchiorum* Hemsl.	Herbaceous parts	Li et al. (2017)
*Gentiana pneumonanthe* L.	Herbaceous parts	Popovic et al. (2021)
*Gentiana crassicaulis* Duthie ex Burk	Dried roots	Liang et al. (2013b)
*Gentiana siphonantha* Maxim. ex Kusnez.	Rhizomes and roots	Tan and Kong (1997)
*Gentiana loureirii* (G.Don) Griseb.	Whole botanical drugs	Wu et al. (2009)
*Gentiana tibetica* King ex Hook. f.	Roots	Tan et al. (1998)
*Gentiana scabra* Bunge	Roots and rhizomes	He et al. (2015)
*Gentiana lutea* L. subsp. aurantiaca	Roots	González-López et al. (2024)
*Cornus officinalis* Sieb. et Zucc.	Dried mature fruit peel	Cai et al. (2013)
*Lonicera caerulea* Linn.	Fruits	Szumny et al. (2024)
*Guettarda speciosa* Linn.	Leaves	Tan et al. (2019)
*Lonicera japonica* Thunb.	Flower buds	Woo et al. (2019)
*Blackstonia perfoliata* (L.) Huds.	Whole botanical drugs	Mihailovic et al. (2020)
*Centaurium erythraea* Rafn	Leaves	Guedes et al. (2019)
*Swertia nervosa* (G. Don) Wall. ex C. B. Clarke	Roots, stems, leaves	Li et al. (2018)
*Neopicrorhiza scrophulariiflora* (Pennell) D. Y. Hong	Rhizomes	Wu et al. (2023)
*Plumeria rubra* Linn.	Stem bark	Alhozaimy et al. (2017)
*Lonicera japonica* Thunb.	Stems	Zhang et al. (2024)
*Swertia bimaculata* (Siebold and Zucc.) Hook.f. and Thomson ex C.B.Clarke, *Swertia cordata* (G.Don) Wall. ex C.B.Clarke, *Swertia paniculata* Wall., *Halenia elliptica* D.Don	Aerial parts	Singh et al. (2019)
*Lonicera japonica* Thunb., *Lonicera similis* Hemsl.	Flower buds or early flowers	Yang et al. (2023)
*Lomatogonium rotatum* (L.) Fr.	Whole botanical drugs	Dai et al. (2023)
*Swertia corymbosa* (Griseb.) Wight ex C.B. Clarke	Aerial parts	Mahendran and Bai (2014)
*Dipsacus asper* Wall.	Roots	Tao et al. (2016)
*Lonicera japonica* Thunb.	Stems	Zhang et al. (2015)
*Viburnum lutescens* Blume.	Leaves	Dang et al. (2021)
*Lonicera hypoglauca* Miq.	Stems and leaves	Yao et al. (2021)
*Nauclea officinalis* (Pierre ex Pit.) Merr. and Chun	Stems, branches, leaves, bark	Zhou et al. (2021b)
*Zeltnera beyrichii* (Torr. and A. Gray) Mans.	Aerial parts	Radovic et al. (2013)
*Centaurium erythraea* Rafn.	Whole botanical drugs	Mandova et al. (2017)
*Swertia japonica* Makino.	Whole plant	Chiba et al. (2011)
*Picrorhiza kurroa* Royle ex Benth.	Stems	Win et al. (2019)
*Pauridiantha callicarpoides* (Hiern) Bremek.	Stem bark	Tatuedom et al. (2014)
*Tabernaemontana cymosa* Jacq.	Seeds	Achenbach et al. (1997)
*Alstonia macrophylla* Wall.ex G. Don	Stems	Changwichit et al. (2011)
*Nauclea orientalis* (L.) L.	Roots	Sichaem et al. (2012)
*Weigela subsessilis* (Nakai) L.H.Bailey	Aerial parts	Won et al. (2015)
*Swertia mussotii* Franch.	Whole botanical drugs	Chen et al. (2015)
*Nauclea orientalis* (L.) L.	Stems	Dao et al. (2015)
*Guettarda pohliana* Müll.Arg.	Roots	de Oliveira et al. (2013)
*Chione venosa* (Sw.) Urb., *Chione venosa* var. venosa	Roots	Lendl et al. (2005)
*Gentianella austriaca* (A.Kern. and Jos.Kern.) Holub	Aerial parts, roots, seeds	Popovic et al. (2023)
*Dipsacus fullonum* L.	Leaves, roots	Oszmiański et al. (2020)
*Dipsacus asper* Wall. ex DC.	Roots	Koo et al. (2013)
*Cephalanthus glabratus* (Spreng.) K.Schum.	Leaves	Tomassini et al. (2020)
*Dipsacus asper* Wall. ex DC.	Roots	Tian et al. (2007)
*Alstonia boonei* De Wild.	Stem bark	Adepiti et al. (2024)
*Tripterospermum chinense* (Migo) H. Smith	Aerial parts	Zhu et al. (2007)
*Alstonia rostrata* C. E. C. Fisch.	Stem bark	Keawpradub et al. (1994)
*Cornus mas* Linn.	Fruits	Deng et al. (2013)
*Fagraea fragrans* Roxb.	Bark and leaves	Jonville et al. (2008)
*Anthocleista djalonensis* A. Chev	Roots, stems, leaves	Onocha et al. (1995)
*Pterocephalus pinardii* Boiss.	Aerial parts	Gülcemal et al. (2010)
*Strychnos nux-blanda* Hill	Roots	Sichaem et al. (2016)
*Manulea altissima* L. f.	Whole botanical drugs	Gousiadou et al. (2015)
*Chironia krebsii* Griseb., *Chironia palustris* Burch., and *Chironia baccifera* L.	Roots	Wolfender et al. (1993)
*Scabiosa columbaria* L.	Rhizomes	Horn et al. (2001)
*Anthocleista vogelii* Planch.	Root bark	Anyanwu et al. (2019)
*Lonicera quinquelocularis* Hardw.	Roots	Kumar et al. (2000)
*Helia alata* (Aubl.) Kuntze	Leaves	Sanchez et al. (2013)
*Citronella gongonha* (Mart.) R.A. Howard	Leaves	Damtoft et al. (1993)
*Anthocleista djalonensis* A. Chev.	Roots	Baba and Usifoh (2011)
*Eucnide bartonioides* Zucc.	Whole botanical drugs	Rodriguez et al. (1997)
*Viburnum erosum* Thunb.	Stems	In et al. (2014)

### 3.2 Biosynthetic pathways and regulatory mechanisms of Sweroside

Although the biosynthetic mechanism of Sweroside remains incompletely understood, current research has uncovered critical pathways and regulatory networks, offering valuable insights into its metabolic processes. In plants of the Gentianaceae family, the biosynthetic pathway of secoiridoids involves the sequential transformation of loganic acid into Sweroside, followed by its conversion into gentiopicroside through the intermediary swertiamarin (Li et al., 2018). This pathway was further corroborated by the work of Tovilovic-Kovacevic and colleagues (Tovilovic-Kovacevic et al., 2020), whose findings not only elucidated the metabolic route of Sweroside but also provided a theoretical framework for studying its intricate transformation and accumulation mechanisms within plant systems.

**FIGURE 1:**
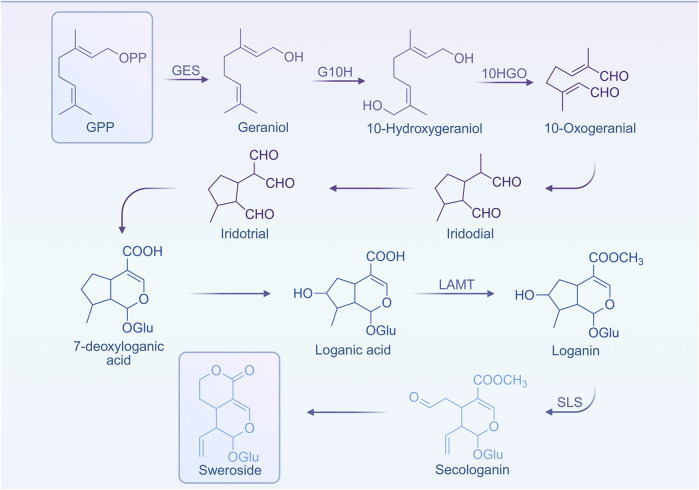
Biosynthetic pathway of Sweroside.

The biosynthesis of terpenoid metabolites fundamentally relies on two primary metabolic pathways: mevalonate pathway (MVA) and the methylerythritol phosphate pathway (MEP, also known as 1-deoxy-D-xylulose-5-phosphate pathway, DXP) pathway. These pathways converge within the metabolic network, jointly facilitating the synthesis of isoprenoid diphosphate (IPP) and dimethylallyl diphosphate (DMAPP)—essential precursors for cyclic terpenoid biosynthesis. Notably, in Sweroside biosynthesis, the majority (up to 95%) of IPP and DMAPP is derived via the MEP pathway (Wang et al., 2001). Starting from these precursors, geranyl diphosphate (GPP) is formed, which, through a series of enzyme-catalyzed reactions and intermediate transformations, gives rise to loganic acid, the direct precursor to Sweroside. The stepwise elucidation of this pathway provides critical direction for future exploration into the biosynthesis of Sweroside (Figure 1).

In addition to this, the accumulation of secondary metabolites in plants often demonstrates notable organ and tissue specificity. For instance, in the aerial reproductive structures of Gentiana pneumonanthe, Sweroside constitutes the predominant metabolite (Popovic et al., 2019). Similarly, in Swertia mussotii, the distribution of secoiridoid glycosides varies distinctly across different tissues. Interestingly, studies have revealed an apparent disconnect between the expression patterns of key biosynthetic genes and the spatial variability in metabolite distribution, suggesting the involvement of highly complex regulatory systems (Liu et al., 2017). Factors such as metabolic enzyme activity, substrate availability, and the cellular microenvironment likely contribute to these nuanced distribution patterns, thereby presenting niche for future investigations into underlying regulatory mechanisms. Additionally, recent studies indicate that WRKY transcription factors may play a pivotal role in the biosynthesis of secoiridoids, exhibiting close associations with the expression of GmWRKY genes. For example, treatment with methyl jasmonate (MeJA) led to increased expression of GmWRKY genes and enzymes implicated in Sweroside biosynthesis. However, paradoxically, this did not significantly enhance Sweroside content (Yin et al., 2024), suggesting the existence of unidentified regulatory mechanisms. Potential factors may include enzyme activity modulation, metabolic network crosstalk, or sophisticated regulation of signaling pathways.

Although the biosynthetic pathways and regulatory mechanisms underlying Sweroside remain incompletely mapped, existing studies have laid a solid groundwork for unraveling its molecular basis and metabolic architecture. These advances not only provide a scientific foundation for optimizing industrial extraction and production processes of Sweroside but also serve as a springboard for exploring the biosynthesis and metabolic engineering of other secoiridoid metabolites.

## 4 Pharmacological activities of sweroside: mechanisms and pathway insights

### 4.1 Anti-inflammatory potential of sweroside: unraveling its molecular targets

Nuclear Factor kappa-light-chain-enhancer of activated B cells (NF-κB) is a pivotal transcription factor that orchestrates the expression of pro-inflammatory cytokines, such as IL-6 and TNF-α, and plays a central role in the inflammatory response (Wang B. et al., 2024). Numerous studies have demonstrated that Sweroside exerts potent anti-inflammatory effects by significantly reducing the release of pro-inflammatory cytokines, including IL-6 and IL-8, through the inhibition of NF-κB activity. This mechanism positions Sweroside as a promising therapeutic agent for various inflammatory diseases (Jiang et al., 2014). For instance, in the context of cardiovascular diseases, Sweroside effectively suppresses inflammation in cardiomyocytes by modulating the CaMKIIδ/NF-κB/NLRP3 signaling pathway. Specifically, Sweroside directly binds to CaMKIIδ, mitigating ROS-mediated inflammatory signaling induced by Angiotensin II (Ang II). This action inhibits the activation of the NF-κB/NLRP3 inflammasome, significantly alleviating heart failure caused by cardiac overload (Wang D. et al., 2024). Similarly, Sweroside reduces inflammation-induced damage during myocardial ischemia-reperfusion by inhibiting NF-κB/NLRP3 activation (Li et al., 2021).

Notably, Sweroside also enhances its anti-inflammatory effects through the activation of the deacetylase SIRT1. This activation leads to reduced expression of inflammation-related enzymes, such as iNOS and COX-2, and a decrease in pro-inflammatory cytokines, including TNF-α, IL-1β, and IL-6. Concurrently, Sweroside promotes the production of the anti-inflammatory cytokine IL-10 (Wang et al., 2019). This dual action of inhibiting pro-inflammatory mediators while enhancing anti-inflammatory responses underscores its therapeutic potential in treating conditions such as osteoarthritis, heart failure, and diabetic nephropathy. In animal models, Sweroside has been shown to alleviate acute lung injury (ALI) and pulmonary edema induced by lipopolysaccharide (LPS). This effect is achieved by upregulating SIRT1 expression, reducing NF-κB p65 and IκBα activation, and downregulating TNF-α and IL-1β secretion. Importantly, inhibition of the SIRT1 pathway markedly diminishes the protective effects of Sweroside, further validating the critical role of SIRT1 activation in its anti-inflammatory mechanism (Wang et al., 2021). These findings provide a novel therapeutic strategy and molecular basis for managing inflammatory diseases, including pneumonia.

In addition to its role in cardiovascular and pulmonary conditions, Sweroside displays substantial anti-inflammatory and bone-regulating effects in osteoarthritis and rheumatoid arthritis. Studies have shown that Sweroside dose-dependently inhibits NF-κB activation in MC3T3-E1 osteoblasts by reducing IKKα and p-NF-κB protein levels while upregulating IκB expression. This regulation enhances mineralized nodule formation and increases the expression of osteoblast-related markers, such as RUNX2, COL1A1, OCN, and OPG, while simultaneously inhibiting RANKL expression. Furthermore, Sweroside mitigates LPS-induced osteoblast inflammation by reducing TNF-α, IL-1β, and IL-6 secretion and activating the PI3K/AKT/GSK-3β/β-catenin signaling pathway (Wei L., 2021). This dual action promotes osteoblast mineralization and inhibits excessive osteoclast differentiation. In rheumatoid arthritis models, Sweroside has been shown to induce apoptosis in fibroblast-like synoviocytes by suppressing Bcl-2 expression and reducing osteoclast formation (Wu et al., 2021). This evidence highlights its potential as a therapeutic agent for regulating bone metabolism and mitigating inflammation in arthritis.

Beyond the NF-κB pathway, Sweroside also exerts anti-inflammatory effects by inhibiting the mTORC1 signaling pathway. This inhibition reduces the production of nitric oxide (NO) and prostaglandin E2 (PGE2) induced by IL-1β, as well as the expression of matrix metalloproteinases (MMP-1, MMP-3, MMP-13) and ADAMTS-5 mRNA (Zhang et al., 2019). In a rat model of chronic liver injury induced by CCl4, Sweroside was administered at doses ranging from 75 to 250 mg/kg. Moreover, no toxicity was observed within the experimental dose range, and 125 mg/kg significantly alleviated hepatic inflammation and promoted liver repair. Histological analysis showed a reduction in inflammatory cell infiltration and alleviation of inflammatory damage in liver tissue. And *in vitro* experiments, Sweroside reduced the source of inflammatory cells by inhibiting the proliferation of HSCs, thereby alleviating the inflammatory response in liver tissue (Gong et al., 2020). Additionally, Sweroside inhibits the activity of key inflammatory enzymes, including COX-2, iNOS, and 5-lipoxygenase (5-LOX), further contributing to its anti-inflammatory effects (Ryu et al., 2010). As a natural secoiridoid metabolites, Sweroside achieves its anti-inflammatory activity through diverse pathways and targets. For example, studies have shown that Sweroside inhibits cyclooxygenase (COX-1 and COX-2) activity, offering a safer alternative to nonsteroidal anti-inflammatory drugs (NSAIDs) due to its lower toxicity. Sweroside, extracted from Pterocephalus hookeri, has demonstrated significant anti-inflammatory effects (Wang et al., 2019). These extracts regulate the expression of iNOS and COX-2, inhibit pro-inflammatory cytokines (e.g., TNF-α, IL-1β, and IL-6), and show therapeutic efficacy in rheumatoid arthritis models.

**FIGURE 2:**
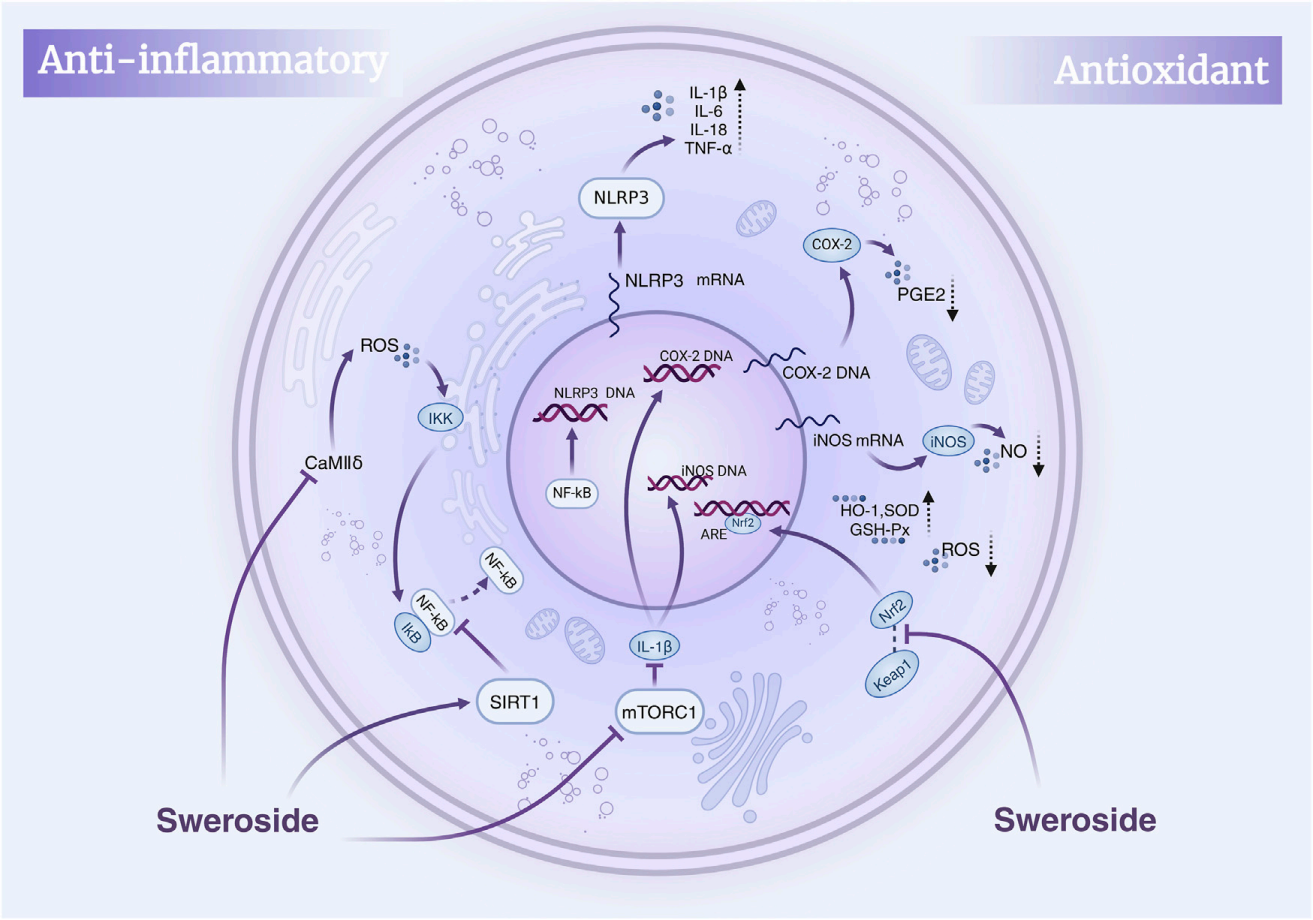
Mechanisms of Sweroside’s anti-inflammatory and antioxidant effects. Sweroside suppresses inflammatory factors by inhibiting NF-κB and activating SIRT1, thereby exerting anti-inflammatory actions. Concurrently, it activates Nrf2 to enhance antioxidant enzyme expression and inhibits iNOS and COX-2, leading to reduced ROS and NO production and robust antioxidant activity.

Collectively, this body of evidence positions Sweroside as a natural anti-inflammatory metabolite with extensive effects mediated through the NF-κB and related signaling pathways, as well as other molecular targets. Its ability to regulate multiple pathways highlights its therapeutic potential in treating inflammatory diseases, including liver disorders, osteoporosis, and cardiovascular conditions. These insights provide a robust molecular foundation for the development of novel anti-inflammatory drugs based on Sweroside (Figure 2).

### 4.2 Antioxidant properties of sweroside: bridging cell protection with therapeutic potential

Sweroside also shows remarkable antioxidant properties. Its capacity to inhibit crucial enzymes and modulate multiple biological pathways clearly demonstrates this. Studies have identified Sweroside in Schenkia spicata, where its antioxidant and enzyme-inhibitory potential has been evaluated through both *in vitro* experiments and computer-aided molecular docking studies. Specifically, Sweroside demonstrates notable inhibitory effects on butyrylcholinesterase (BChE), tyrosinase, α-amylase, and glucosidase. Molecular docking analyses reveal that Sweroside interacts with BChE and tyrosinase via hydrogen bonding and van der Waals forces, highlighting its potential applications in neuroprotection. Similarly, its binding to α-amylase and glucosidase elucidates the molecular mechanisms underlying its anti-diabetic enzyme activity. Chemical assays, including the CUPRAC and FRAP methods, further confirm its moderate antioxidant capacity, with Sweroside facilitating the reduction of Cu^2+^ to Cu^+^ (21.14 mg TE/g) and Fe^3+^ to Fe^2+^ (12.32 mg TE/g) (Zengin et al., 2023). Although both methods showed certain reducing abilities, they cannot fully represent their antioxidant effects in biological systems. And the molecular docking results may not completely correspond to the inhibition rates obtained from actual experiments, and further *in vivo* and *in vitro* experiments are needed to confirm this. Additionally, Sweroside has been shown to mitigate DNA damage induced by 4-nitroquinoline one-oxide (4-NQO), demonstrating significant anti-genotoxic activity (Deng et al., 2013). This protective effect is largely attributed to its antioxidant properties, offering new avenues for the prevention and treatment of genotoxicity-related diseases (Cvetkovic et al., 2020).

Excessive production of reactive oxygen species (ROS) is a key driver of oxidative stress and inflammation, and Sweroside has demonstrated potent efficacy in regulating ROS levels. Quantitative analyses of *Cornus officinalis* fruit extracts, which are rich in Sweroside, have revealed its ability to significantly inhibit ROS production. Furthermore, water-based extracts containing Sweroside effectively suppressed the release of tumor necrosis factor-α (TNF-α) by neutrophils, an effect attributed to the synergistic action of Sweroside and other bioactive metabolites. Sweroside is one of the bioactive components in Cornus extract, and the extract enriched in sweroside exhibits enhanced antioxidant and immunomodulatory properties (Czerwinska et al., 2021). In myocardial protection studies, Sweroside has shown strong protective effects against oxidative stress-induced damage. It significantly reduces ROS and malondialdehyde (MDA) accumulation, thereby inhibiting lipid peroxidation, while simultaneously increasing the activity of key antioxidant enzymes, such as superoxide dismutase (SOD) and glutathione peroxidase (GSH-Px). Moreover, Sweroside induces the expression of heme oxygenase-1 (HO-1) and promotes the activation and nuclear translocation of Nrf2 by inhibiting the Keap1-Nrf2 interaction, thereby enhancing cellular antioxidant responses. This evidence underscores Sweroside’s regulatory role in the Nrf2-dependent Keap1/Nrf2 axis (Li et al., 2021), offering a novel mechanism for mitigating ischemia-reperfusion injury, improving myocardial viability, and reducing the release of injury biomarkers.

Oral administration of high concentrations of Sweroside extract has been shown to enhance the endogenous antioxidant defense system. This is evidenced by increased SOD and catalase (CAT) activity, elevated glutathione (GSH) levels, and a marked reduction in MDA (Dang et al., 2021). In an animal model of oxidative stress induced by CCl_4_, Sweroside, as the primary active bioactive metabolites of Gentiana asclepiadea extract, significantly enhanced catalase and SOD activity, increased reduced glutathione levels, and decreased MDA content (Tao et al., 2019). These biochemical changes highlight Sweroside’s ability to bolster the oxidative stress defense system, providing robust evidence for its potential as a therapeutic antioxidant agent.

## 5 Sweroside in translational medicine and therapeutic applications

### 5.1 Hepatoprotective effects of sweroside: mechanistic insights and clinical implications

The liver, the largest visceral organ in the human body, performs an array of vital physiological functions. However, liver injury can initiate a cascade of health complications, some of which may become life-threatening. In recent years, mounting evidence has highlighted the therapeutic potential of Sweroside in liver protection, owing to its robust anti-inflammatory and antioxidant properties. Early studies from the 1990s revealed that the n-butanol (n-BuOH) extract of Swertia, which Sweroside included, exhibits significant hepatoprotective effects against D-galactosamine (D-GalN)/lipopolysaccharide (LPS)-induced liver injury in mice (Hase et al., 1997). Subsequent investigations expanded its application scope to conditions like non-alcoholic steatohepatitis (NASH). In a NASH mouse model induced by a methionine-choline-deficient (MCD) diet, intraperitoneal administration of Sweroside significantly suppressed the activation of the NLRP3 inflammasome in primary macrophages, leading to a reduction in IL-1β and caspase-1 production. This intervention not only attenuated serum aspartate aminotransferase (AST) and alanine aminotransferase (ALT) levels but also reduced immune cell infiltration, triglyceride accumulation, and fibrosis in liver tissues. Furthermore, Sweroside inhibited NLRP3 inflammasome activation by obstructing the *de novo* synthesis of mitochondrial DNA, intensifying its hepatoprotective effects. The study also employed the MTT assay to assess the cytotoxicity of Sweroside on cells, and it was observed that treatment with Sweroside up to 100 μM did not induce significant cytotoxicity. (Yang G. et al., 2020). Collectively, these advances underscore Sweroside’s anti-inflammatory and antioxidant efficacy in various liver injury models. But the study only observed the short-term effects of Sweroside and cannot determine the impact of long-term Sweroside treatment on the pathological process. In addition to inflammation, fat accumulation, and fibrosis, the pathological features of NASH also include insulin resistance, oxidative stress, etc. The study failed to evaluate the effect of Sweroside on these pathological features, so it is impossible to fully understand the role of Sweroside in the overall pathological process of NASH.

Non-alcoholic fatty liver disease (NAFLD), a complex metabolic liver disorder, is closely linked to heightened liver-related morbidity and mortality and is a precursor to other diseases, including type 2 diabetes (Ballestri et al., 2016) and atherosclerosis (Mikolasevic et al., 2016). The activation of peroxisome proliferator-activated receptor α (PPAR-α) emerges as a critical mechanism in mitigating NAFLD by promoting anti-inflammatory gene expression and suppressing inflammatory mediators (Bougarne et al., 2018). In a study using C57BL/6 mice (HFD) model, Sweroside treatment at doses of 120 mg/kg and 240 mg/kg significantly reduced weight gain, improved glucose tolerance and insulin sensitivity, mitigated hepatic steatosis, lowered serum lipid levels, and diminished liver inflammation. These effects were strongly associated with the activation of PPAR-α (Yang Q. L. et al., 2020). Moreover, *in vitro* analyses revealed that the main metabolite of Sweroside in the rats’ body, Sweroside aglycone (Sweroside-M1), could activate PPAR-α transcription, offering an additional avenue for its therapeutic effects on NAFLD. By leveraging this dual mechanism, Sweroside opens new possibilities for NAFLD treatment. The duration of the trial was 6 weeks, which may not be sufficient to investigate the long-term efficacy and potential chronic toxicity of Sweroside in NAFLD.

Bile acid regulation is pivotal within the enterohepatic system, yet excessive bile acid accumulation in the liver can directly or indirectly induce hepatocyte injury (Chatterjee et al., 2014). Recent findings have highlighted Sweroside’s protective role in cholestatic liver diseases (Malhi et al., 2010). In ANIT-induced C57BL/6J mice model, mice treated with Sweroside (120 mg/kg), the compound significantly downregulated the expression of bile acid synthesis-related genes (e.g., CYP7A1, CYP8B1, and HSD3B7) while concurrently inhibiting the expression of bile acid transporters, such as Mrp2, Ost-β, Mdr1, NTCP, Oatp1a1, and Oatp1b2 (Yang et al., 2016). This regulatory activity diminished pro-inflammatory responses and alleviated liver-specific injuries. In this experiment, Sweroside markedly reduced the expression of pro-inflammatory markers and normalized elevated serum ALT and AST levels, alleviating hepatocyte necrosis. Additionally, it reversed the increase in serum alkaline phosphatase (ALP), total bilirubin (TBIL), direct bilirubin (DBIL), and total bile acids (TBA) caused by ANIT exposure, improving liver-function indicators. Importantly, these investigations reported no observable toxicity for Sweroside in either cellular or animal models, further substantiating its safety profile as a promising therapeutic candidate. In liver protection mechanisms, cytochrome P450 (CYP450) enzyme regulation represents a pivotal area of focus (Dai et al., 2018). Specifically, CYP3A4—the most abundant CYP isoenzyme in the liver—is instrumental in bile acid metabolism and drug detoxification. Studies suggest that CYP3A4 also has therapeutic implications in mitigating aconitine-induced toxicity. HepG2 cell studies demonstrated that amarogentin exhibits strong binding affinity to CYP3A4, inducing its mRNA expression, thereby expediting aconitine metabolism and reducing its hepatotoxicity. Although Sweroside’s binding score is lower than that of Amarogentin, it is higher than that of some other compounds such as Loganic acid and Swertiamarin, indicating that it may have certain potential in regulating CYP3A4. However, the study was only conducted in HepG2 cells, with a lack of *in vivo* experimental data. Moreover, there are no existing studies that have deeply explored the mechanism of action of Sweroside, such as its impact on CYP3A4 protein expression and enzyme activity. And future studies need to verify the efficacy and safety of Sweroside in animal models, further investigate its mechanism of action.

**FIGURE 3:**
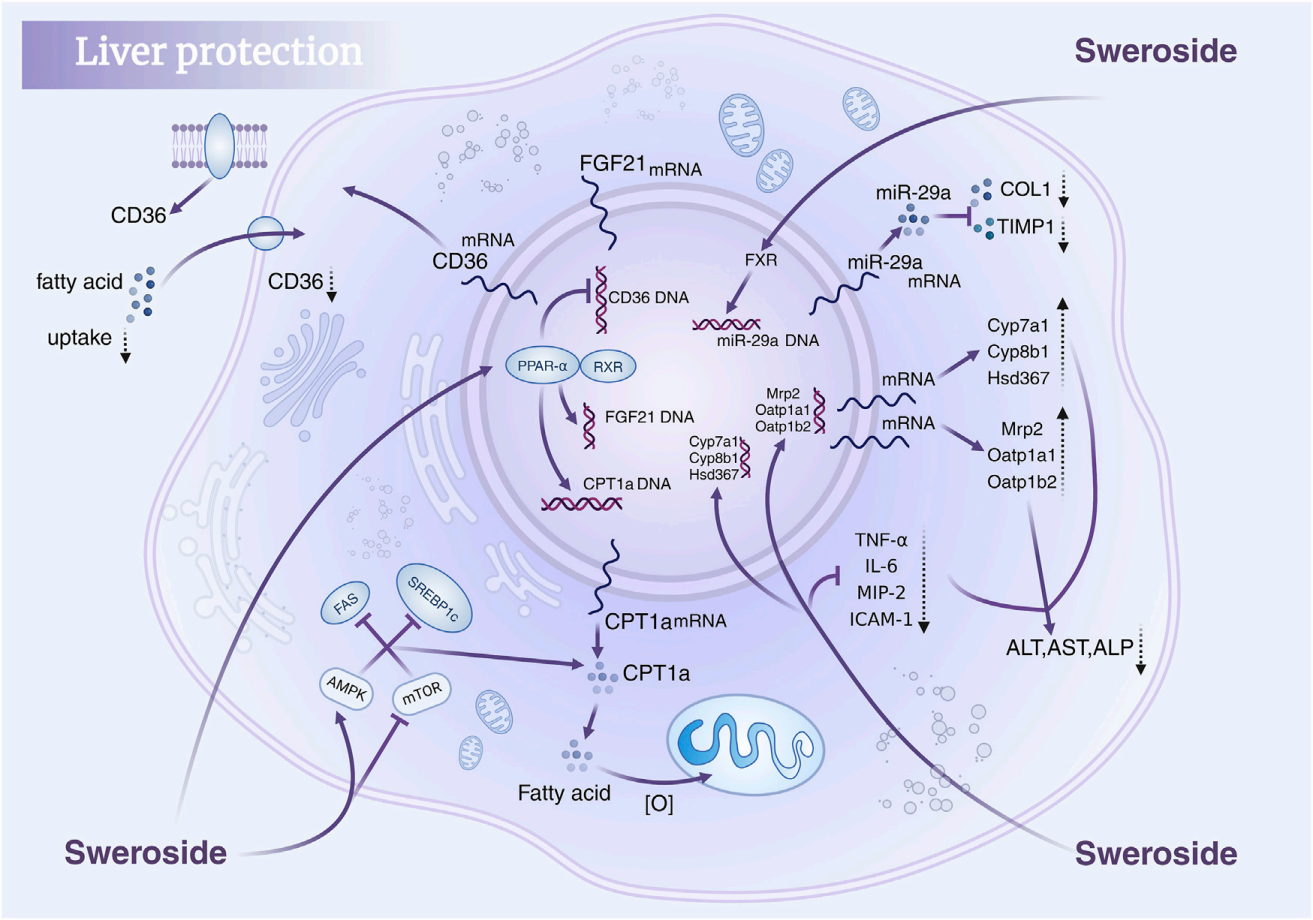
Hepatoprotective mechanisms of Sweroside. It regulates key signaling pathways, modulates gene expression, reduces fatty acid uptake, suppresses inflammatory responses, and lowers markers of liver injury, thereby protecting liver health.

In addition, Sweroside’s hepatoprotective potential is further supported by findings from studies on Gentianaceae plant extracts rich in Sweroside. Extracts from the aerial parts and roots of *Gentiana asclepiadea* L. (Mihailovic et al., 2013b) and *Gentiana cruciata* L. (Mihailović et al., 2014) increased CAT, SOD, and GSH levels in a CCl_4_-induced liver injury model while significantly reducing MDA levels, thereby ameliorating oxidative-stress-induced liver damage. Similarly, in Gentianella turkestanerum, the butanol extract, with its high secoiridoid glycoside content, exhibited superior efficacy in alleviating acute liver injury compared to other extracts (Yang et al., 2017). These extracts reversed reductions in SOD, GSH, and CAT activity in a dose-dependent manner, further corroborating their hepatoprotective properties. In summary, Sweroside exhibits multifaceted therapeutic potential in liver-disease models, functioning through anti-inflammatory, antioxidant, bile acid regulatory, and mitochondrial DNA synthesis enhancement mechanisms. These findings not only underscore the value of Sweroside as a drug candidate but also present innovative strategies for the clinical management of liver diseases, paving the way for novel therapeutic interventions (Figure 3).

### 5.2 Sweroside in osteoporosis management: stimulating bone formation and repair

Osteoporosis, a prevalent metabolic bone disease, is characterized by an imbalance between bone formation and resorption, with osteoblast proliferation and differentiation playing a pivotal role in its treatment (Almeida et al., 2017; Christenson, 1997). Sex steroid hormones and their receptors are intimately linked to bone metabolism, and maintaining this delicate balance is essential for effective therapeutic strategies. Sweroside has demonstrated significant potential in regulating bone metabolism through multiple mechanisms. Studies have identified Sweroside as a key active metabolite in the traditional Chinese medicine Xianlinggubao Capsule (XLGB), where it activates adenylate cyclase (AC) to promote the biosynthesis of sex steroid hormones. By enhancing hormone synthesis, Sweroside contributes to the maintenance of bone health and the prevention of osteoporosis (Tang et al., 2021).

Beyond its endocrine regulatory effects, the antioxidant properties of Sweroside further bolster its anti-osteoporotic efficacy. Extracts of *Gentiana macrophylla* Pall., rich in Sweroside, have exhibited potent antioxidant activity in DPPH, NBT, and FRAP assays, with IC_50_ values of 275, 262, and 180 μg/mL, respectively, in a concentration-dependent manner. These extracts also reduced serum ALP levels, decreased urinary calcium excretion, and increased serum calcium concentrations, suggesting a protective effect against osteoporosis mediated by antioxidant mechanisms. Sweroside, as a key bioactive metabolite of these extracts, likely contributes to these observed benefits, further emphasizing its multifaceted role in bone health (Gong et al., 2018). Sweroside’s ability to promote osteoblast proliferation and differentiation adds another dimension to its therapeutic potential. In studies using Sweroside extracted from *Cornus officinalis* fruits, the metabolite significantly enhanced the proliferation of human MG-63 cells and rat osteoblasts (P < 0.01) without exhibiting cytotoxicity at concentrations of 10^–5^ and 10^–6^ g/mL. Furthermore, Sweroside treatment increased ALP activity in MG-63 cells and rat osteoblasts by approximately 1.4- and 1.3-fold (P < 0.05), respectively, and significantly elevated osteocalcin secretion compared to control groups (P < 0.05). These findings indicate that Sweroside directly promotes osteoblast differentiation, suggesting that its anti-osteoporosis effects stem from its ability to enhance osteogenic function (Sun et al., 2013). In the experiment, two cell models, human MG-63 cells and rat osteoblasts, were used to comprehensively evaluate the osteogenic effect of Sweroside by MTT assay, ALP activity assay, osteocalcin secretion assay and flow cytometry. However, there is a lack of *in vivo* experiments, making it difficult to evaluate the long-term effects of drugs.

Mechanistic studies have further elucidated Sweroside’s osteogenic efficacy. By activating the p38 signaling pathway through interactions with membrane estrogen receptor-α and GPR30, Sweroside enhances the differentiation and mineralization efficiency of MC3T3-E1 pre-osteoblasts (Wu et al., 2020). The limitations of this study include the absence of *in vivo* experiments and a lack of long-term efficacy assessment like the experiments conducted by Sun et al. (2013) Nevertheless, both experiments provide robust evidence supporting the potential therapeutic application of Sweroside in osteoporosis treatment. The findings from the first experiment offer a more comprehensive evaluation across multiple parameters, whereas the current study delves deeper into elucidating the underlying molecular mechanisms of action. Additionally, Sweroside regulates the expression of key proteins and transcription factors involved in osteogenesis. In an ovariectomized (OVX) mouse model, Sweroside significantly upregulated the expression of osteogenesis-related genes, including RUNX2, OSX, OCN, Collagen I, BMP2, and OPN, by activating the mTORC1/PS6 signaling pathway. The most pronounced effects were observed at a concentration of 1 μg/mL. This mechanism facilitates the differentiation of bone marrow mesenchymal stem cells (BMSCs) into osteoblasts, effectively mitigating menopause-related osteoporosis (Ding et al., 2019). Furthermore, Sweroside promotes the formation of mineralized bone matrix by regulating the BMP2/CBFA1 signaling pathway, providing critical support for bone repair and the generation of new bone tissue (Choi et al., 2021).

**FIGURE 4:**
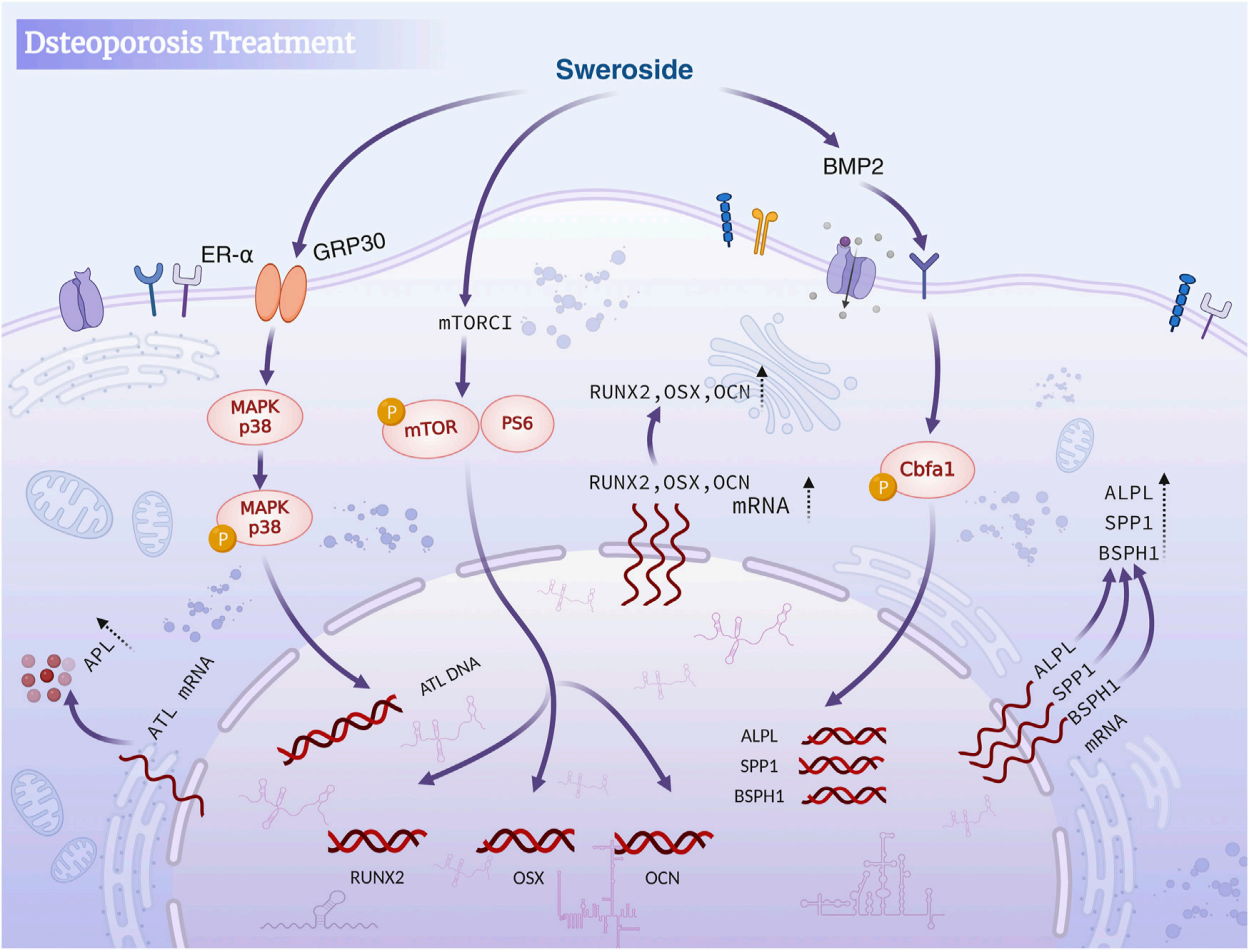
Sweroside in osteoporosis treatment. The compound activates the BMP2 signaling pathway and mTORC1 to inhibit osteoclast activity, promote osteoblast differentiation and maturation, enhance bone formation, and reduce bone resorption, ultimately aiding in the treatment of osteoporosis.

Studies have indicated that Sweroside may exert anti-osteoporotic effects through multiple mechanisms, such as regulating endocrine function, enhancing antioxidant activity, promoting the proliferation and differentiation of osteoblasts, and inhibiting their apoptosis, which are mediated by the activation of key signaling pathways. Most existing studies are limited to *in vitro* cell experiments, lacking in-depth investigations into its long-term efficacy and mechanism of action *in vivo*. Future research should include more *in vivo* experiments, clarify its targets and signal transduction mechanisms, so as to facilitate the application of Sweroside in the treatment of osteoporosis. In brief, Sweroside exerts its anti-osteoporotic effects through a multi-targeted approach, including antioxidant mechanisms, direct promotion of osteoblast proliferation and differentiation, and regulation of key signaling pathways such as mTORC1 and BMP2/CBFA1. These works establish Sweroside as a promising natural metabolite with significant potential for the prevention and treatment of osteoporosis, offering a comprehensive strategy to address the multifactorial nature of this disease (Figure 4).

### 5.3 Sweroside as an anti-cancer agent: targeting tumor growth and pathway regulation

Sweroside has garnered considerable attention for its multi-target anti-cancer properties. Inhibiting tumor cell proliferation, inducing cell cycle arrest and apoptosis, and suppressing tumor growth *in vivo* are all manifestations of anti-cancer activity (Chen et al., 2024). This section explores the mechanisms by which Sweroside regulates cell proliferation, apoptosis, and signaling pathways, while highlighting its potential application in cancer therapy. Sweroside exerts its anti-cancer effects by modulating key signaling pathways involved in tumor metabolism and growth. In a liver cancer model, Sweroside and gentiopicroside exhibited synergistic anti-cancer effects by jointly activating the Akt and Erk1/2 signaling pathways (Huang et al., 2016). This activation resulted in the suppression of phosphoenolpyruvate carboxykinase 1 (Pck1), a metabolism-related enzyme that is frequently overexpressed in various cancers. The combination of these compounds significantly enhanced the activity of Akt and Erk1/2 signaling, amplifying the inhibitory effects on tumor cells. By reducing Pck1 levels, Sweroside disrupts tumor metabolism, a mechanism of considerable importance for therapeutic strategies targeting metabolic disorders in cancer.

Beyond its metabolic regulation, Sweroside’s ability to modulate the cell cycle and induce apoptosis forms the core of its anti-cancer mechanisms. Studies on acute myeloid leukemia (AML) revealed that Sweroside significantly inhibited the proliferation of human leukemia cell lines *in vitro*, with half-maximal inhibitory concentration (IC_50_) values ranging from 62.58 to 318.60 µM. Sweroside exhibits significant anti-leukemia activity in the range of 40–80 µM (*in vitro*) and 50–100 mg/kg (*in vivo*), with its mechanism of action achieved through dual pathways of cell cycle arrest and apoptosis. Importantly, Sweroside exhibited lower toxicity toward normal cell lines, establishing a favorable safety profile for its clinical application. Mechanistic investigations showed that Sweroside induced S and G2/M phase cell cycle arrest in HL-60 leukemia cells by downregulating cell cycle regulatory proteins Cyclin D1, CDK4, and CDC2, while upregulating cell cycle inhibitors p53 and p21. This dual effect effectively halts leukemia cell proliferation. Concurrently, Sweroside activated multiple apoptosis-related pathways, increasing the expression of Cleaved Caspase-9, Caspase-3, and PARP, while reducing the anti-apoptotic protein Bcl-2 and upregulating the pro-apoptotic protein Bax (Han et al., 2017). This cascade of molecular events triggers programmed cell death, thereby blocking further tumor progression.

Similar mechanisms have been observed in solid tumor studies. For instance, the methanol extract of Gentiana kurroo roots, which is rich in Sweroside, exhibited potent anti-cancer effects against pancreatic cancer cells (Miapaca-2). This extract induced G0/G1 phase cell cycle arrest and triggered programmed cell death, demonstrating Sweroside’s comprehensive regulation of the cell cycle and apoptosis (Wani et al., 2013). In glioblastoma (U251 cell) models, Sweroside showed high anti-proliferative activity, mediated by its inhibition of the JNK/p38 MAPK signaling pathway. This pathway is critical for cancer cell proliferation and survival. Sweroside also enhanced the expression of pro-apoptotic proteins Caspase-3, Caspase-9, and Bax, while suppressing the anti-apoptotic protein Bcl-2, thereby promoting apoptosis in glioblastoma cells (Ouyang and Xu, 2019). The 5–20 µM range shows significant anti-tumor activity against U251 glioblastoma cells. The IC50 of Sweroside for U251 cells (10 µM) is much lower than that for normal astrocytes (100 µM), supporting its targeting. Since this study is only *in vitro* data, additional animal model validation is needed to confirm *in vivo* efficacy. In addition to inhibiting cancer cell proliferation, Sweroside also effectively suppresses cancer cell metastasis. It achieves this by modulating the Wnt/β-catenin signaling pathway, thereby inhibiting the transcriptional activity of β-catenin and downregulating the expression of downstream target genes, including c-myc, Cyclin D1, Survivin, and MMP-7. Furthermore, Sweroside reduces the ability of cancer cells to form spheroids and colonies while decreasing the expression of stem cell markers CD133 and CD44. These actions collectively inhibit cancer cell proliferation, invasion, and migration, and block the acquisition of stem cell-like properties (Huang et al., 2021). As a result, Sweroside holds significant potential as a therapeutic agent for prostate cancer (PCa) bone metastasis. These findings underscore Sweroside’s potential as a therapeutic agent for highly invasive tumors.

**FIGURE 5:**
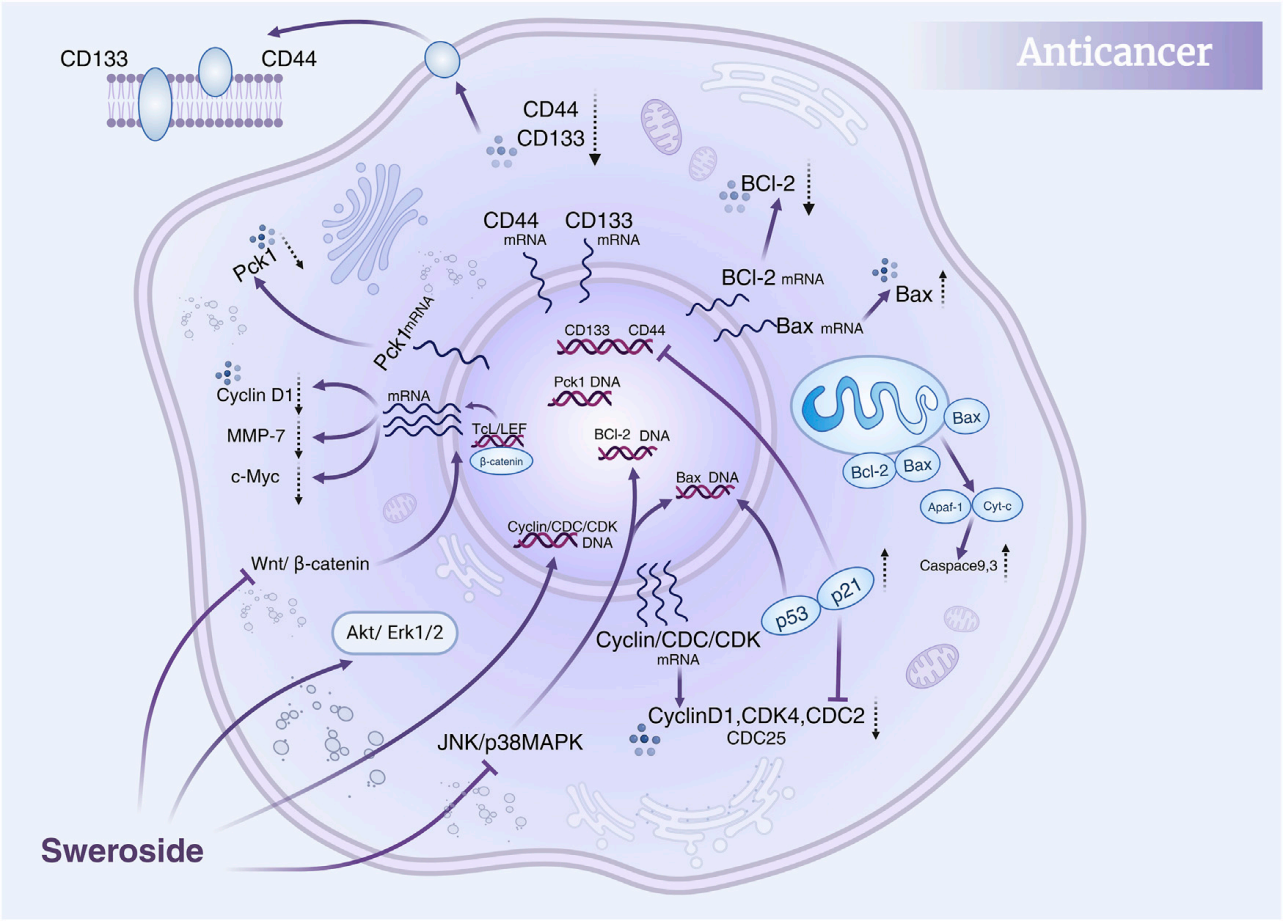
Anti-cancer mechanisms of Sweroside. It modulates the Akt/Erk1/2, JNK/p38 MAPK, and Wnt/β-catenin signaling pathways, regulates the cell cycle, and inhibits cancer cell proliferation and migration. Furthermore, it promotes apoptosis, contributing to its anti-cancer efficacy.

Collectively, these studies highlight the multidimensional anti-cancer activity of Sweroside, which encompasses direct regulation of the cell cycle, promotion of apoptosis, and fine-tuning of multiple signaling pathways (Figure 5). Moreover, the combination of Sweroside with other metabolites, such as gentiopicroside, has been shown to amplify its anti-cancer effects through synergistic interactions. This suggests that Sweroside’s therapeutic potential extends beyond its use as a standalone agent, offering valuable insights into combination therapies and targeted interventions. Sweroside has demonstrated significant anti-tumor potential in various cancer models, exerting its effects by inhibiting cell proliferation, inducing apoptosis, arresting the cell cycle, and regulating signaling pathways. Existing studies lack long-term *in vivo* experimental data, the mechanism of action remains incompletely clarified, and drug resistance has not been evaluated. Future research should conduct long-term *in vivo* experiments to assess its long-term efficacy and toxicity, and explore its mechanism of action in depth. Additionally, attempts can be made to conduct combined studies with other multi-target drugs to evaluate whether it can overcome drug resistance or enhance therapeutic effects.

### 5.4 Sweroside in diabetic nephropathy: improving podocyte function and glucose metabolism

Diabetic nephropathy (DN), a leading chronic complication of diabetes, arises from a multifaceted pathogenesis involving oxidative stress, inflammation, metabolic dysregulation, and apoptosis. Recent studies have highlighted Sweroside as a promising therapeutic agent for DN, demonstrating protective effects through diverse mechanisms and offering new avenues for the treatment of diabetes and its associated complications. One of Sweroside’s key therapeutic actions is its ability to protect podocytes from high glucose (HG)-induced injury and apoptosis. In C57BL/6 mice model of DN, 120 mg/kg/day by gavage for 3 months, Sweroside significantly improved podocyte structure and function, stabilizing the glomerular filtration barrier by upregulating nephrin and podocin expression. Concurrently, it downregulated desmin and hypoxia-inducible factor-1α (HIF1α), markers associated with pathological damage, thereby mitigating DN-induced functional impairments. Moreover, Sweroside alleviated proteinuria, glomerular injury, and metabolic disturbances by activating the Akt/BAD signaling pathway. Specifically, it increased the expression of the pro-survival protein BAD and phosphorylated Akt (p-Akt), shielding podocytes from HG-induced damage and apoptotic signals (Huang et al., 2023).

In addition to its protective effects on podocytes, Sweroside exhibits potent anti-inflammatory and antioxidant properties. By modulating the SIRT1/NF-κB signaling pathway (Ma et al., 2023), Sweroside at 100 μM upregulated SIRT1 expression, facilitating the deacetylation of the p65 NF-κB subunit and thereby reducing inflammatory damage in HK-2 proximal tubular epithelial cells. This mechanism highlights Sweroside’s ability to attenuate inflammation by targeting key inflammatory signaling pathways. Oxidative stress is also one of the causes of diabetes and its complications (Darenskaya et al., 2021). Future studies should incorporate animal models to provide a more comprehensive assessment of Sweroside’s therapeutic potential for DN. Furthermore, Sweroside demonstrated robust antioxidant activity, reducing oxidative stress levels and inhibiting free radical production. These effects further alleviate oxidative stress-induced cellular damage, providing a strong theoretical basis for its role in mitigating diabetes-related complications. Beyond its renal protective properties, Sweroside also plays a critical role in regulating glucose metabolism. Gluconeogenesis, a key metabolic pathway contributing to hyperglycemia in diabetes, is driven by Pck1, an enzyme central to blood glucose regulation. Studies have shown that Sweroside, alone or in combination with gentiopicroside, significantly suppresses Pck1 expression in HL1C cells, mimicking insulin-like effects by inducing the phosphorylation of Akt and Erk1/2 signaling pathways (Huang et al., 2016). This synergistic mechanism underscores the potential of Sweroside and gentiopicroside in regulating gluconeogenesis and addressing metabolic dysregulation. Additionally, Sweroside has demonstrated broader metabolic regulatory effects. For instance, in the chloroform fraction of *Anthocleista vogelii* root bark, Sweroside exhibited dose-dependent inhibition of α-amylase and α-glucosidase activity, improving glucose tolerance in obese diabetic animal models and increasing serum insulin levels (Anyanwu et al., 2019).

Taken together, Sweroside may exerts protective effects in diabetic nephropathy through multiple mechanisms, including the regulation of podocyte injury, modulation of key signaling pathways, and improvement of glucose metabolism. Its ability to reduce oxidative stress and inflammatory damage further enhances its therapeutic potential as a multi-target natural metabolite. Future studies could investigate the specific molecular mechanisms underlying the therapeutic effects of Sweroside in diabetic nephropathy (DN) from aspects such as antioxidant activity, anti-inflammatory effects, and regulation of cellular signaling pathways, as well as its differential roles in various renal cell types. Additionally, research could be conducted on the efficacy of combining Sweroside with other kidney disease treatment methods to explore its potential value in comprehensive therapy.

### 5.5 Cardioprotective effects of sweroside: a multi-pathway approach to myocardial health

Sweroside, a naturally derived metabolite with a unique cyclopentane core, possesses marked biological activity and holds significant promise in alleviating a spectrum of pathological myocardial injuries (Zhang et al., 2020). Its robust antioxidant and anti-inflammatory properties, particularly within the cardiovascular system, have garnered increasing attention. Recent investigations have uncovered the vital role of Sweroside in myocardial protection, achieved through multifaceted regulatory mechanisms targeting inflammation, oxidative stress, apoptosis, and metabolic dysregulation.

In the context of myocardial ischemia/reperfusion (MI/R) injury, the NLRP3 inflammasome plays a pivotal role in driving myocardial damage through pyroptosis, an inflammatory form of programmed cell death (Mezzaroma et al., 2021). Studies have demonstrated that Sweroside effectively inhibits the expression of NLRP3, ASC, IL-1β, and Caspase-1, thus suppressing the activation of the inflammasome and mitigating pyroptosis. This mechanism significantly reduces cardiomyocyte injury in MI/R models (Li et al., 2021). Furthermore, in a chronic heart failure mouse model induced by isoproterenol (ISO), Sweroside alleviated myocardial energy metabolism disturbances by upregulating Na^+^/K^+^-ATPase (ATP1A1) expression, indicating its potential to improve cardiomyocyte function by modulating metabolic processes (Qing et al., 2019). These researches suggest that Sweroside exerts protective effects in myocardial injury by synergizing anti-inflammatory and metabolic regulatory mechanisms. Innovatively, combining Sweroside with exosomes derived from mesenchymal stem cells (MSCs) has been shown to amplify its cardioprotective effects. This combined therapy, particularly in sepsis-induced myocardial damage, demonstrated superior efficacy in reducing oxidative stress, apoptosis, and inflammatory responses, underscoring the therapeutic advantage of such integrative approaches (Wang et al., 2023). Additionally, in experimental models of pressure overload (TAC/Ang II-induced myocardium damage), Sweroside effectively inhibited the CaMKIIδ/NF-κB/NLRP3 signaling axis, further illustrating its capacity to target upstream regulators of inflammation and apoptosis in myocardial injury (Wang D. et al., 2024). This study used *in vitro* cell models and *in vivo* mouse models, and used a variety of experimental methods to rigorously reveal the mechanism of Sweroside’s improvement of heart failure. Compared to other cardioprotective effects experiments, this experiment has positive controls. Sweroside demonstrates comparable efficacy to the positive control drug Captopril in improving cardiac function and alleviating myocardial fibrosis. However, it exhibits potential advantages in anti-inflammatory effects, direct targeting of CaMKIIδ, multi-target mechanisms, and its origin as a natural product.

The cardioprotective properties of Sweroside extend to mitigating aconitine-induced myocardial toxicity. Aconitine exposure in H9c2 cardiomyocytes elicits pathological hallmarks such as oxidative stress, calcium overload, mitochondrial dysfunction, autophagy dysregulation, and apoptosis. Sweroside’s treatment effectively inhibited the production of intracellular ROS induced by Aconitine and significantly downregulated the mRNA levels of LC3-II, Beclin-1, and Caspase-3 induced by Aconitine. Experimental evidence indicates that Sweroside restores calcium homeostasis by modulating the expression of Ca^2+^-handling proteins, including NaV1.5, RyR2, DHPR, and SERCA, thereby preventing calcium overload and its downstream pathological consequences. Moreover, Sweroside safeguards mitochondrial membrane potential (ΔΨ), stabilizing intracellular calcium dynamics and mitigating aconitine-induced cell injury (Ma et al., 2018). Sweroside’s ability to suppress autophagy and apoptosis induced by Aconitine further highlights its significance as a potential therapeutic agent for myocardial injuries arising from exogenous toxins. This study provides valuable insights into the mechanisms underlying the cardioprotective effects of Sweroside. Sweroside also demonstrates efficacy in counteracting pathological myocardial hypertrophy, a key contributor to the progression of numerous cardiovascular diseases. This pathological condition is tightly linked to the activation of the Akt/mTOR/HIF-1α signaling axis. By inhibiting this pathway, Sweroside attenuates the hypertrophic response, marked by reductions in H9c2 cardiomyocyte surface area and suppression of hypertrophy-associated markers such as ANP and β-MHC. Additionally, it decreases the expression of phosphorylated Akt, mTOR, and HIF-1α, thereby preventing pathological myocardial remodeling through metabolic reprogramming and pathway regulation (Chen et al., 2021).

**FIGURE 6:**
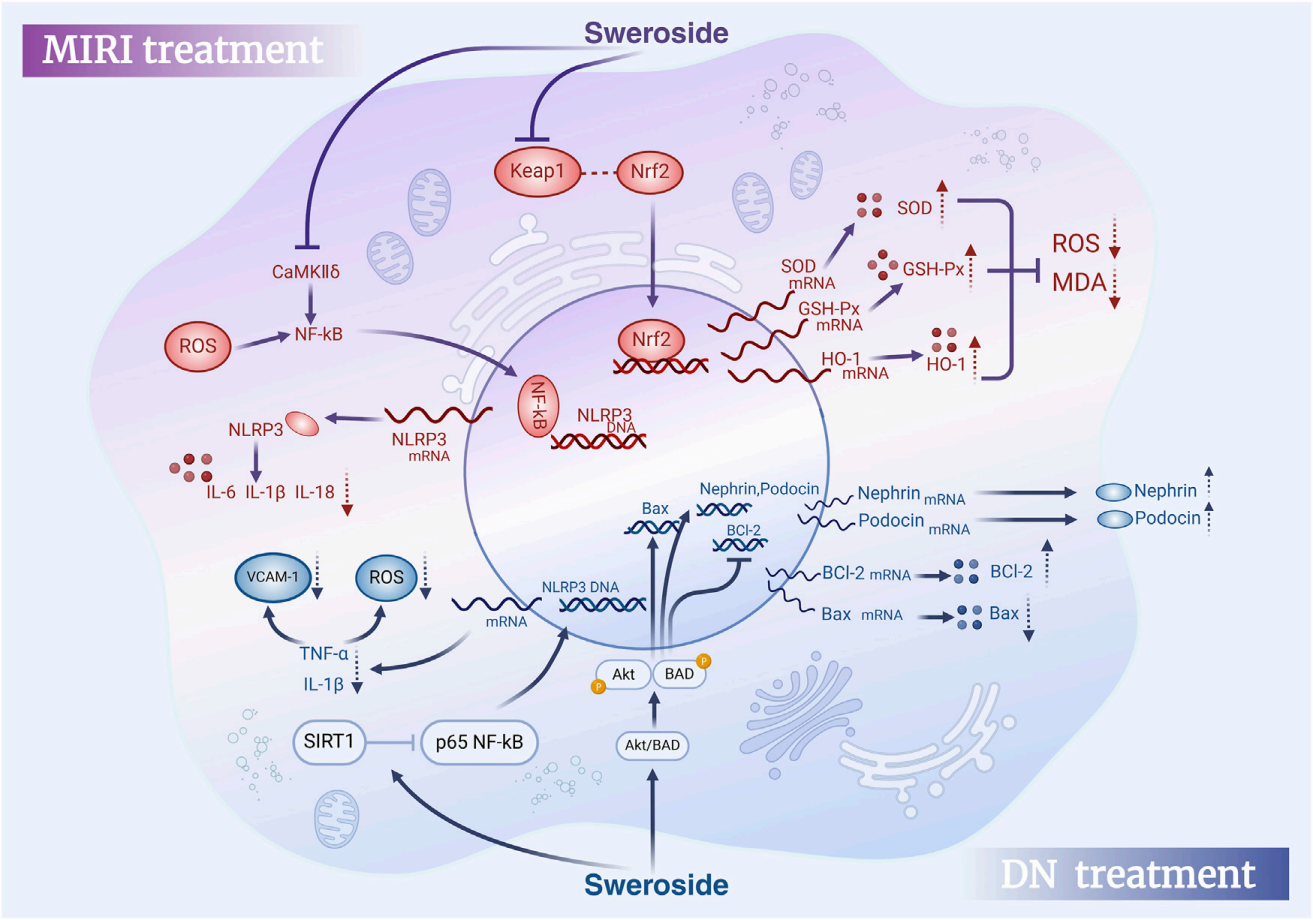
Therapeutic effects of Sweroside in MIRI and DN. In myocardial ischemia-reperfusion injury (MIRI), Sweroside activates the Nrf2 signaling pathway to promote antioxidant enzyme expression and reduce pro-inflammatory factor release. In diabetic nephropathy (DN), it activates the Akt/BAD signaling pathway, modulates Bcl-2 family proteins, Nephrin, and Podocin expression, and decreases pro-inflammatory factor release, offering therapeutic benefits.

To sum up, Sweroside exhibits potent cardioprotective effects through a variety of biological mechanisms, including modulation of inflammatory and pyroptosis signaling pathways, improvement of energy metabolism, restoration of calcium homeostasis, and reversal of pathological myocardial remodeling. These findings highlight the ability of Sweroside to alleviate myocardial hypertrophy and heart failure, as well as its potential for preventing and treating MIRI and DICT. The accumulated evidence provides a robust theoretical foundation for exploring the clinical applications of Sweroside in cardiovascular medicine. However, future research should integrate detailed mechanistic studies with clinical trials to fully elucidate the therapeutic potential of this multifaceted compound (Figure 6).

### 5.6 Neuroprotection and dermatological applications of sweroside: from brain health to skin disorders

Sweroside shows notable therapeutic potential across diverse disease domains, including metabolic, cardiovascular, neurodegenerative, and dermatological conditions. Its multidimensional biological activities, especially in neuroprotection and skin pathology regulation, highlight its promise as a candidate for novel therapeutic applications.

Sweroside has demonstrated notable neuroprotective effects by modulating various pathological mechanisms underlying neurodegenerative diseases. *In vitro* studies using PC12h cells revealed that Sweroside significantly promotes neurite outgrowth, a crucial marker of nerve regeneration. Its effects are concentration-dependent, with neurite outgrowth at 100 µM comparable to that induced by nerve growth factor (NGF) (Chiba et al., 2011). These findings suggest that Sweroside holds promise as a therapeutic candidate for the prevention and treatment of neurodegenerative disorders. Behavioral studies further corroborate its neuroprotective potential. In scopolamine-induced *Danio rerio* models of cognitive impairment, Sweroside significantly improved anxiety and memory deficits, the Sweroside-treated group increased risk-taking behavior, enhanced novelty preference, and altered locomotor activity, as demonstrated through the novel water maze test (NTT), Y-maze test, and novel object recognition test (NOR). Mechanistically, Sweroside inhibited acetylcholinesterase (AChE) activity, thereby enhancing cholinergic neurotransmission. Concurrently, it boosted the activity of antioxidant enzymes, including SOD, catalase (CAT), and glutathione peroxidase (GPX), while increasing reduced GSH levels and reducing protein carbonylation (Brinza et al., 2022). Whether Sweroside has clinical relevance requires further validation in mouse models, and this study offers a hypothetical basis for future animal experiments. The neuroprotective effects of Sweroside require further evaluation in mouse models, and this study provides a hypothetical basis for future animal experiments. Another hallmark of AD pathology is the abnormal aggregation of Tau protein, which is closely linked to neuronal loss and cognitive decline. Studies have shown that Sweroside, as the primary secoiridoid glycoside in *Cornus officinalis*, significantly reduces the accumulation of Tau oligomers (Xu et al., 2020). This effect underscores its potential to slow disease progression and supports its candidacy as a therapeutic agent for AD. Sweroside also exhibits favorable safety profiles in central nervous system applications. In studies involving methanol extracts from Gentiana roots, Sweroside, along with other active metabolites such as gentiopicroside and swertiamarin, synergistically enhanced swimming endurance in mice. At doses of 250 and 500 mg/kg, the average swimming times increased from 10.5 min in the control group to 15.8 and 16.2 min, respectively. Additionally, Sweroside demonstrated mild analgesic activity in the tail-clip test and exhibited no significant toxicity at high doses. This evidence highlights its safety and efficacy, further evaluating its potential as a therapeutic metabolite for nervous system disorders (Özturk et al., 2002).

In addition to its neuroprotective properties, Sweroside has shown considerable promise in regulating skin health, particularly through its effects on pigment metabolism. Melanin, a pigment synthesized from tyrosine metabolism, plays a protective role against ultraviolet radiation. However, excessive melanin accumulation can lead to pigmentation disorders. Studies have demonstrated that Sweroside, at a concentration of 300 μM, significantly inhibits melanin production in melan-a cells without inducing cytotoxic effects (Saito et al., 2024). This inhibition is achieved by downregulating tyrosinase, a key enzyme in melanin biosynthesis, along with its associated proteins TRP-1 and TRP-2. Further, studies using zebrafish models revealed that Sweroside activates Akt and ERK in a dose-dependent manner, leading to reduced pigmentation and tyrosinase activity. These findings suggest that Sweroside has the potential to be developed as a skin-whitening agent and a therapeutic candidate for treating pigment-related skin disorders (Jeong et al., 2015).

Overall, the diverse biological activities of Sweroside, including its antioxidant, anti-inflammatory, and Tau protein aggregation-reducing effects in neurodegenerative diseases, as well as its enzyme-regulatory activity and safety in skin pigment metabolism, provide a strong theoretical foundation for its application across multiple disease domains (Table 2). Future research should focus on elucidating its mechanisms of action in complex pathological environments, optimizing its dosage forms and delivery strategies, and advancing its development as a multi-target therapeutic agent. These efforts could pave the way for novel treatments addressing neurodegenerative disorders, skin pigmentation abnormalities, and beyond.

**TABLE 2:** Overview of Sweroside’s therapeutic effects across diverse disease models.

Diseases	*In-vitro*/vivo models	Dose/Administration form	Mechanisms/Effects	Key findings	References
Hepatic Disease	*In vivo*: C57BL/6 mice HFD-induced NAFLD animal model	60, 120, 240 mg/kg, for 6 weeks	PPAR-α is activated, CD36↑ and FGF21↓	Modulation of lipid metabolism and inflammatory response to improve fatty liver associated with obesity	Yang et al. (2020b)
	*In vivo*: C57BL/6 mice non-alcoholic fatty liver disease (NAFLD) model; *In vitro*: primary mouse hepatocytes	120 mg/kg/day, for 3 months; 1 µg/m L for 24h	Partially activate autophagy via the AMPK/mTOR signaling pathway	Partial activation of autophagy via the AMPK/mTOR signaling pathway as an effective hepatic autophagy activator	Ding et al. (2023)
	*In vivo*: mouse model of liver fibrosis induced by CCl_4_.	125 mg/kg/day (i.g.), for 7 days	miR-29a↑, COL1 and TIMP1↓	Regulation of the FXR-miR-29a axis to adjust FXR expression and transcriptional activity, exerting an anti-fibrotic effect	Gong et al. (2020)
	*In vivo*: NASH mouse model	30 mg/kg (i.p.)	Inhibition of NLRP3 inflammasome activation IL-1β and caspase-1↓	Anti-inflammatory activity mediated by blocking the formation of the NLRP3 inflammasome complex.	Yang et al. (2020a)
	*In vivo*: C57BL/6J mice	120 mg/kg/day (i.g.), for 5 days	β-MCA, CA, and TCA ↓ Reduced expression of pro-inflammatory cytokine mRNA and protein TNF-α, IL-6, mKC, MIP-2, and ICAM-1 ↓	Restoring bile acid synthesis and transporting it to normal levels, while inhibiting pro-inflammatory responses, effectively alleviates ANIT-induced cholestatic liver injury in mice.	Yang et al. (2016)
Osteoporosis	*In vitro*: MG-63 cells and rat osteoblasts	7.5 mg/L	ALP activities Osteocalcin ↑	Exerting a direct osteogenic effect, promoting the proliferation and differentiation of MG-63 cells and rat osteoblasts *in vitro*.	Sun et al. (2013)
	*In vitro*: MC3T3-E1 cell	1 μM	interact with membrane estrogen receptor-α and GPR30 Activate the p38 signaling pathway	Alkaline phosphatase (ALP) activity is promoted, and osteoblast mineralization is increased in MC3T3-E1 cells, without affecting their proliferation.	Wu et al. (2020)
	*In vivo*: OVX mice; *In vitro*: BMSCs	120 mg/kg/day, for 3 months; 0.1, 1, 10 μg/mL	Expression of P-mTOR, PS6, RUNX2, OSX, and cyanide ↑ Differentiation of osteoblasts ↑	Excessive activation of the mTORC1/PS6 signaling pathway, effectively inhibits OP.	Ding et al. (2019)
	*In vivo*: OVX mice	0.01, 0.1, and 1 µM	BMC and BMD levels ↑ Bone marrow adipocytes ↓ BMP2 and RUNX2 ↑ ALPL, SPP1 and BSPH1 ↑	Regulation of BMP2/RUNX2-mediated molecules, inducing the formation of mineralized bone matrix for osteoporosis treatment by generating new bone matrix.	Choi et al. (2021)
	*In vitro*: RAW264.7 cell	20, 40, and 80 μM, for 5 days	NF-κB ↓ FOXO1 signaling pathway ↑	Improving LPS-induced inflammation in RAW264.7 cells by activating SIRT1 and regulating the SIRT1/NF-κB and SIRT1/FOXO1 signaling pathways.	Wang et al. (2019)
	*In vitro*: Rat chondrocytes	0.1, 1, 10 µg/mL	IL-1β-induced NO and PGE2 ↓ MMP-1, MMP3, MMP13, and ADAMTS-5 mRNA ↓ NF-κB ↓ and Iκ-B ↑ Phosphorylation of S6K1 and S6 ↓	Inhibition of NF-κB and mTORC1 signaling pathways, mediating anti-inflammatory activity.	Zhang et al. (2019)
Cancer	*In vitro*: U251 glioblastoma and normal human astrocytes	0, 5, 10, 20 µM for 24 h	JNK/p38 MAPK signaling ↓ Bax ↑, Bcl-2 ↓ Caspase-3 and Caspase-9 ↑	Inducing U251 cells to arrest in the G0/G1 phase of the cell cycle exerts a potent anticancer effect on glioblastoma cells.	Ouyang and Xu (2019)
	*In vitro*: PC-3 cells	0, 5, 10, 20 µM for 24 h	Induction of apoptosis and intracellular ROS Apoptotic proteins ↑ Spherical formation, colony formation, and expression of CD133 and CD44 ↓ TTCF/LEF activity ↓ c-Myc, surviving, MMP-7 ↓ Catenin transcription ↓	Anti-tumor effect in the development of prostate cancer bone metastasis.	Huang et al. (2021)
	*In vivo*: NU/NU mice; *In vitro*: HL - 60 cells	0, 20, 40, 80 µM for 24 h; Mice dosages: 25, 50, 100 mg/kg	Cyclin D1, CDK4, CDC2 and CDC25↓ p53 and p21↑ Caspase - 3 and PARP↑ Bcl-2 ↓, Bax↑	Induction of S and G2/M cell-cycle arrest and inhibition of human leukemia cell proliferation, emerging as a promising candidate drug for preventing human leukemia progression.	Han et al. (2017)
Diabetic Nephropathy	*In vitro*: HK - 2 cells	0, 25, 50, 100 µM	Levels of TNF - α, IL - 1β and VCAM - 1↓ Level of ROS↓ Degree of EMT↓ Level of SIRT1↑ Acetylation of p65 NF - κB↓	Alleviating the damage of HG - 2 cells by promoting SIRT1 - mediated deacetylation of p65 NF - κB.	Ma et al. (2023)
	*In vivo*: C57BL/6 mouse DN model; *In vitro*: MPC-5	120 mg/kg/day, for 3 months (i.g.); 1 µg/mL	Podocyte apoptosis↓ Expressions of renin and podocin↑ Expressions of Desmin and HIF1α↓	Stimulating the activation of the Akt/BAD pathway, regulating Bcl-2 - associated death promoter (BAD) and p - Akt, and improving HG - induced podocyte injury and apoptosis.	Huang et al. (2023)
	*In vivo*: TAC mice and Ang II - infused mice; *Invitro*: H9c2, AC16 and NRCM	0.01, 0.1, 1, 10, 100 µg/mL for 24 h; Dosages: 15, 30, 60 mg/kg/day	It directly binds to CaMKIIδ in cardiomyocytes, thereby inhibiting the ROS - mediated NF - κB/NLRP3 pathway. Levels of IL - 1β, IL - 18 and IL - 6↓	Having a significant inhibitory effect on TAC/AngII - induced CaMKIIδ/NF - κB/NLRP3 in mouse cardiomyocytes.	Wang et al. (2024b)
Cardiovascular Disease	*In vivo*: C57/BL6N mouse heart failure model	120 mg/kg/day, for 40 consecutive days.	Expression of ATP1A1↑	Significantly improving isoproterenol (ISO)-induced heart failure in mice. Its mechanism may be related to increasing the expression of ATP1A1 protein and improving myocardial energy metabolism.	Qing et al. (2019)
	*In vivo*: aconitine - induced arrhythmia rat model; *In vitro*: H9c2 cells	2, 10, 20 μM; 50 mg/kg/day, for 5 days	Aconitine - induced mRNA expressions of NaV1.5 (encoded by SCN5A), RyR2, and DHPR↓, mRNA of SERCA↑ It prevents the continuous accumulation of intracellular Ca^2+^ and avoids intracellular Ca^2+^ overload. mRNA expressions of Beclin - 1, Caspase - 3, and LC3 - II↓	Protecting H9c2 cells from aconitine - induced autophagy and apoptosis, and reducing aconitine - induced arrhythmia.	Ma et al. (2018)
	*In vivo*: Wistar rats; vitro: H9c2 cells	0, 5, 10, 25, 50, 100 μM, for 24 h; Mice dosages: 25, 50, 100 mg/kg	CK - MB and LDH↓ Reactive oxygen species and malondialdehyde↓ Activities of superoxide dismutase and glutathione peroxidase↑ Activities of caspase - 1 and interleukin (IL) - 1β↓ NLRP3↓ Apoptosis - associated speck - like protein↓ Caspase - 1 and IL - 1β↓ Keap1↓ Induced nuclear translocation of nuclear factor erythroid 2 - related factor 2 (Nrf2)	Sweroside pretreatment can protect against myocardial ischemia - reperfusion (IR) injury by partially inhibiting oxidative stress and NLRP3 inflammasome - mediated pyroptosis through regulating the Keap - 1/Nrf2/ROS axis.	Li et al. (2021)
	*In vitro*: PC12h cells	1, 5, 10, 20, 50, 100 μM, for 2 days	Induced neurite outgrowth	Inducing neurite outgrowth in PC12h cells.	Chiba et al. (2011)
Neurological Disorders	*In vitro*: Zebrafish model	2.79, 8.35, 13.95 nM, for 8 days	Acetylcholinesterase activity↓ Brain oxidative stress↓	Having a significant impact on anxiety and cognitive impairment, which is partly driven by regulating cholinergic system activity and brain antioxidant effects. It is a potential anti - amnesia candidate drug for zebrafish.	Brinza et al. (2022)
	*In vivo*: LPS-induced acute lung injury (ALI) model in mice	15, 30, 60 mg/kg (i.p.)	NF - κB signaling pathway↓ Expression of SIRT1↑	Protecting LPS - induced ALI mice by inhibiting inflammatory effects.	Wang et al. (2021)
Others	Melan - a cells	150, 300 µM	Protein levels of 346 MITF, phosphorylated Akt and ERK↓, Melanogenesis↓	Acting as an effective skin - lightening agent by regulating the expression of 40 MAP kinases and melanogenic enzymes, and could potentially be used as a natural depigmenting agent.	Jeong et al. (2015)
	Molecular docking		It shows enzyme inhibitory effects on butyrylcholinesterase (BChE), tyrosinase, α- amylase and glucosidase.	Having potential antidiabetic and neuroprotective activities.	Zengin et al. (2023)

## 6 Pharmacokinetics and advanced delivery strategies for sweroside

Sweroside, recognized for its potent anti-inflammatory and antioxidant properties, has garnered significant research attention. However, pharmacokinetic studies reveal critical limitations, including low oral bioavailability and a short systemic residence time, primarily influenced by its absorption, distribution, metabolism, and excretion characteristics. Current findings elucidate these challenges while offering potential strategies for optimization. The oral bioavailability of Sweroside is notably low, reported at merely 0.31% (Luo et al., 2009). In rat studies, following oral administration of 500 mg/kg, the majority of the metabolite was excreted unabsorbed via feces, accounting for 43.38% ± 3.2% of the dose. This poor absorption is attributed to its high hydrophilicity, which impairs its ability to traverse the intestinal epithelium and enter systemic circulation. The intestinal absorption of Sweroside primarily occurs through passive diffusion and active diffusion. Efflux transporters such as P-glycoprotein (P-gp), multidrug resistance-associated protein (MRP), and breast cancer resistance protein (BCRP) typically restrict the absorption of drugs (Xu et al., 2020). Additionally, the presence of abundant β-glucosidase in human feces accelerates its degradation, further reducing absorption efficiency (Chang et al., 2022). The first-pass metabolism effect compounds this issue, as partial deactivation occurs in the liver during gastrointestinal absorption.

Beyond absorption, the distribution profile of Sweroside is characterized by rapid plasma clearance and limited systemic retention. Intravenous administration of 1 mg/kg in rats revealed a rapid decline in plasma concentrations, equilibrating within 120 min, with a half-life of 33.9 ± 5.2 min and an absolute bioavailability of 11.90% ± 1.33%. Organ distribution studies indicate that Sweroside predominantly accumulates in the kidneys, liver, spleen, and lungs, with minimal penetration across the blood-brain barrier (BBB). This restricted BBB permeability suggests limited potential for central nervous system (CNS) applications. Comparatively, oral administration (5, 10, 15 mg/kg) resulted in delayed plasma concentration peaks (within 60 min) and a slightly extended half-life of 74.5 ± 12.3 min. However, the combined effects of delayed absorption and rapid degradation further constrained its systemic exposure (Bozunovic et al., 2018). Metabolic studies provide additional insights into Sweroside’s pharmacokinetics, highlighting its susceptibility to rapid biotransformation. Using UHPLC/Q-TOF-MS, researchers identified key metabolic pathways, including reduction, N-heterocyclization, N-acetylation, deglycosylation, and glucuronidation (Han et al., 2014). In a gastrointestinal perfusion rat model, Sweroside was rapidly converted into metabolites naucledal and epinaucledal, with naucledal exhibiting significantly higher plasma exposure (threefold greater AUC). This suggests that naucledal may represent the primary active metabolite (Li et al., 2024). Furthermore, *in vitro* studies demonstrate Sweroside’s high sensitivity to β-glucosidase, as enzymatic treatment with almond β-glucosidase nearly eliminated its concentration. These findings underscore the role of external factors, such as enzymatic degradation, in limiting its bioavailability. Excretion studies further emphasize Sweroside’s rapid clearance. Following intravenous administration, plasma concentrations peaked at 6.3 ± 0.4 μM within 30 min, with an elimination half-life (t1/2) of 0.4 ± 0.1 h and a mean residence time of 0.7 ± 0.1 h. The plasma clearance rate was notably high at 0.6 ± 0.1 L/h/kg, with the majority of the compound excreted via urine (fe-U: 80.3% ± 24.6%). A high plasma unbound fraction (fu: 95.7%) further supports the rapid renal excretion of Sweroside, complicating efforts to maintain therapeutic plasma concentrations (Cheng et al., 2016).

Collectively, Sweroside’s pharmacokinetic limitations—low absorption efficiency, rapid distribution, early metabolism, and efficient excretion—result in suboptimal bioavailability and short systemic residence time (Table 3). Despite these challenges, potential solutions are emerging. Prodrug strategies and advanced delivery systems, such as nanotechnology, could enhance intestinal absorption and improve systemic exposure. Additionally, the pharmacological properties of metabolites, particularly naucledal and epinaucledal, warrant further investigation. Future research should focus on isolating and evaluating these metabolites, elucidating their therapeutic potential, and exploring the influence of the intestinal microenvironment on Sweroside metabolism. By addressing these pharmacokinetic barriers, Sweroside could overcome its current limitations and expand its therapeutic applications while retaining its natural pharmacological properties.

**TABLE 3:** Metabolic profile and characteristics of sweroside.

Metabolic site	Metabolic process	Detection method	Relevant data	Main findings	Reference
Intestine Liver Kidney	Most are excreted directly through feces without being absorbed. Uptaken by the liver and excreted via bile. Rapidly excreted through renal tubules.	HPLC - UV Microdialysis technique	Oral bioavailability is 0.31%.	Low oral bioavailability (0.31%); relatively poor intestinal absorption efficiency; high bile concentration, likely closely associated with its hepatoprotective effect.	Luo et al. (2009)
Liver and kidney	Glucuronidation, sulfation, methylation, reduction metabolism by carbonyl reductase (CR), or involvement of the cytochrome P450 (CYP450) enzyme system	LC-MS/MS	Elimination half - life is 0.4 h. Volume of distribution is 0.3 L/kg. Total clearance is 0.6 L/h/kg, accounting for 8% of cardiac output. Urinary excretion fraction reaches 80.3%. Unbound fraction (fu) in plasma is 95.7%.	Low clearance efficiency, likely related to the metabolic capacity of the liver and kidney. Urine is the primary excretory route, with most of the compound existing in free form, facilitating metabolism and excretion.	Cheng et al. (2016)
Liver Gastrointestinal tract	Hydrolysis to aglycone in the gastrointestinal tract, followed by various metabolic reactions: hydrolysis, hydroxylation, reduction, glucuronide conjugation, sulfation, N-heterocyclization, N-acetylation, etc.	UHPLC/Q-TOF-MS, NMR spectroscopy	The aglycone of Sweroside, which is one of the main metabolites. The glucuronide conjugate of aglycone (1 - O - β - glucuronide aglycone), which is one of the metabolites with the highest content in urine.	Metabolism primarily occurs in the gastrointestinal tract and liver, with potential involvement of gut microbiota in hydrolysis. Aglycone and its metabolites are critical for Sweroside’s hepatoprotective effects. Low oral bioavailability due to absorption in the aglycone form.	Han et al. (2014)
Liver and Gastrointestinal Tract	First-pass metabolism in the liver, mainly excreted in urine, with minor excretion through bile and feces	HPLC–MS/MS with electrospray ionization and multiple reaction monitoring	Elimination half-life (t_1/2_): 67.6–78.8 min; Average absolute bioavailability: 11.90%; Pharmacokinetic characteristics: Linear pharmacokinetics; Excretion: Only 2.68% of the administered dose is excreted in the original form, with 0.67% in bile, 1.55% in urine, and 0.46% in feces.	Extensive hepatic metabolism, rapid distribution to various tissues, and excretion in urine as metabolites. Low bioavailability and high liver distribution suggest significant hepatic metabolic transformation, with distribution closely related to tissue blood perfusion rate.	Sheng et al. (2014)
Intestine Liver Kidney	Most are excreted directly through feces without being absorbed. Uptaken by the liver and excreted via bile. Rapidly excreted through renal tubules.	HPLC - UV Microdialysis technique	Oral bioavailability is 0.31%.	Low oral bioavailability (0.31%); relatively poor intestinal absorption efficiency; high bile concentration, likely closely associated with its hepatoprotective effect.	Luo et al. (2009)
Liver and kidney	Glucuronidation, sulfation, methylation, reduction metabolism by carbonyl reductase (CR), or involvement of the cytochrome P450 (CYP450) enzyme system	LC-MS/MS	Elimination half - life is 0.4 h. Volume of distribution is 0.3 L/kg. Total clearance is 0.6 L/h/kg, accounting for 8% of cardiac output. Urinary excretion fraction reaches 80.3%. Unbound fraction (fu) in plasma is 95.7%.	Low clearance efficiency, likely related to the metabolic capacity of the liver and kidney. Urine is the primary excretory route, with most of the compound existing in free form, facilitating metabolism and excretion.	Cheng et al. (2016)
Liver Gastrointestinal tract	Hydrolysis to aglycone in the gastrointestinal tract, followed by various metabolic reactions: hydrolysis, hydroxylation, reduction, glucuronide conjugation, sulfation, N-heterocyclization, N-acetylation, etc.	UHPLC/Q-TOF-MS, NMR spectroscopy	The aglycone of Sweroside, which is one of the main metabolites. The glucuronide conjugate of aglycone (1 - O - β - glucuronide aglycone), which is one of the metabolites with the highest content in urine.	Metabolism primarily occurs in the gastrointestinal tract and liver, with potential involvement of gut microbiota in hydrolysis. Aglycone and its metabolites are critical for Sweroside’s hepatoprotective effects. Low oral bioavailability due to absorption in the aglycone form.	Han et al. (2014)
Liver and Gastrointestinal Tract	First-pass metabolism in the liver, mainly excreted in urine, with minor excretion through bile and feces	HPLC–MS/MS with electrospray ionization and multiple reaction monitoring	Elimination half-life (t_1/2_): 67.6–78.8 min; Average absolute bioavailability: 11.90%; Pharmacokinetic characteristics: Linear pharmacokinetics; Excretion: Only 2.68% of the administered dose is excreted in the original form, with 0.67% in bile, 1.55% in urine, and 0.46% in feces.	Extensive hepatic metabolism, rapid distribution to various tissues, and excretion in urine as metabolites. Low bioavailability and high liver distribution suggest significant hepatic metabolic transformation, with distribution closely related to tissue blood perfusion rate.	Sheng et al. (2014)
Intestine Liver Kidney	Most are excreted directly through feces without being absorbed. Uptaken by the liver and excreted via bile. Rapidly excreted through renal tubules.	HPLC - UV Microdialysis technique	Oral bioavailability is 0.31%.	Low oral bioavailability (0.31%); relatively poor intestinal absorption efficiency; high bile concentration, likely closely associated with its hepatoprotective effect.	Luo et al. (2009)
Liver and kidney	Glucuronidation, sulfation, methylation, reduction metabolism by carbonyl reductase (CR), or involvement of the cytochrome P450 (CYP450) enzyme system	LC-MS/MS	Elimination half - life is 0.4 h. Volume of distribution is 0.3 L/kg. Total clearance is 0.6 L/h/kg, accounting for 8% of cardiac output. Urinary excretion fraction reaches 80.3%. Unbound fraction (fu) in plasma is 95.7%.	Low clearance efficiency, likely related to the metabolic capacity of the liver and kidney. Urine is the primary excretory route, with most of the compound existing in free form, facilitating metabolism and excretion.	Cheng et al. (2016)
Liver Gastrointestinal tract	Hydrolysis to aglycone in the gastrointestinal tract, followed by various metabolic reactions: hydrolysis, hydroxylation, reduction, glucuronide conjugation, sulfation, N-heterocyclization, N-acetylation, etc.	UHPLC/Q-TOF-MS, NMR spectroscopy	The aglycone of Sweroside, which is one of the main metabolites. The glucuronide conjugate of aglycone (1 - O - β - glucuronide aglycone), which is one of the metabolites with the highest content in urine.	Metabolism primarily occurs in the gastrointestinal tract and liver, with potential involvement of gut microbiota in hydrolysis. Aglycone and its metabolites are critical for Sweroside’s hepatoprotective effects. Low oral bioavailability due to absorption in the aglycone form.	Han et al. (2014)
Liver and Gastrointestinal Tract	First-pass metabolism in the liver, mainly excreted in urine, with minor excretion through bile and feces	HPLC–MS/MS with electrospray ionization and multiple reaction monitoring	Elimination half-life (t_1/2_): 67.6–78.8 min; Average absolute bioavailability: 11.90%; Pharmacokinetic characteristics: Linear pharmacokinetics; Excretion: Only 2.68% of the administered dose is excreted in the original form, with 0.67% in bile, 1.55% in urine, and 0.46% in feces.	Extensive hepatic metabolism, rapid distribution to various tissues, and excretion in urine as metabolites. Low bioavailability and high liver distribution suggest significant hepatic metabolic transformation, with distribution closely related to tissue blood perfusion rate.	Sheng et al. (2014)
Intestine Liver Kidney	Most are excreted directly through feces without being absorbed. Uptaken by the liver and excreted via bile. Rapidly excreted through renal tubules.	HPLC - UV Microdialysis technique	Oral bioavailability is 0.31%.	Low oral bioavailability (0.31%); relatively poor intestinal absorption efficiency; high bile concentration, likely closely associated with its hepatoprotective effect.	Luo et al. (2009)
Liver and kidney	Glucuronidation, sulfation, methylation, reduction metabolism by carbonyl reductase (CR), or involvement of the cytochrome P450 (CYP450) enzyme system	LC-MS/MS	Elimination half - life is 0.4 h. Volume of distribution is 0.3 L/kg. Total clearance is 0.6 L/h/kg, accounting for 8% of cardiac output. Urinary excretion fraction reaches 80.3%. Unbound fraction (fu) in plasma is 95.7%.	Low clearance efficiency, likely related to the metabolic capacity of the liver and kidney. Urine is the primary excretory route, with most of the compound existing in free form, facilitating metabolism and excretion.	Cheng et al. (2016)
Liver Gastrointestinal tract	Hydrolysis to aglycone in the gastrointestinal tract, followed by various metabolic reactions: hydrolysis, hydroxylation, reduction, glucuronide conjugation, sulfation, N-heterocyclization, N-acetylation, etc.	UHPLC/Q-TOF-MS, NMR spectroscopy	The aglycone of Sweroside, which is one of the main metabolites. The glucuronide conjugate of aglycone (1 - O - β - glucuronide aglycone), which is one of the metabolites with the highest content in urine.	Metabolism primarily occurs in the gastrointestinal tract and liver, with potential involvement of gut microbiota in hydrolysis. Aglycone and its metabolites are critical for Sweroside’s hepatoprotective effects. Low oral bioavailability due to absorption in the aglycone form.	Han et al. (2014)
Liver and Gastrointestinal Tract	First-pass metabolism in the liver, mainly excreted in urine, with minor excretion through bile and feces	HPLC–MS/MS with electrospray ionization and multiple reaction monitoring	Elimination half-life (t_1/2_): 67.6–78.8 min; Average absolute bioavailability: 11.90%; Pharmacokinetic characteristics: Linear pharmacokinetics; Excretion: Only 2.68% of the administered dose is excreted in the original form, with 0.67% in bile, 1.55% in urine, and 0.46% in feces.	Extensive hepatic metabolism, rapid distribution to various tissues, and excretion in urine as metabolites. Low bioavailability and high liver distribution suggest significant hepatic metabolic transformation, with distribution closely related to tissue blood perfusion rate.	Sheng et al. (2014)

## 7 Conclusion and future perspectives

### 7.1 Mechanisms of sweroside and its limitations in studies

This review systematically examines the presence of Sweroside in medicinal plants, its biosynthetic pathways, and its physicochemical properties, while providing a comprehensive summary of its pharmacological profile and therapeutic potential. Preclinical studies have demonstrated that Sweroside exerts a broad range of effects, including anti-inflammatory, antioxidant, analgesic, hepatoprotective, anti-diabetic, antibacterial, and anticancer activities. These effects are largely mediated through intricate interactions with key signaling pathways, such as NF-κB, NLRP3, MAPK, PI3K/Akt, Akt/BAD, mTOR, and PPAR-α, as well as regulation of pivotal molecular targets and gene expressions.

In the area of hepatoprotection, Sweroside has shown significant promise. By modulating AMPK and mTOR signaling pathways, it activates autophagy, thereby mitigating hepatic damage. It also reduces liver fibrosis through FXR regulation and attenuates lipid metabolism disturbances and inflammatory responses via PPAR-α activation. Moreover, by suppressing the NLRP3 inflammasome, Sweroside reduces the production of inflammatory cytokines, further reinforcing its hepatoprotective effects. These findings position Sweroside as a potent candidate for combating liver-pathologies. Sweroside also demonstrates compelling effects in the management of osteoporosis, where it promotes osteogenesis and enhances bone health. It facilitates the differentiation of bone marrow mesenchymal stem cells into osteoblasts through activation of the mTORC1/PS6 signaling pathway. Additionally, Sweroside contributes to the formation of a mineralized bone matrix by modulating the BMP2/CBFA1 pathway and exerts anti-inflammatory effects through the SIRT1/NF-κB and SIRT1/FOXO1 signaling pathways. By elevating alkaline phosphatase (ALP) activity and osteocalcin secretion, it significantly enhances bone mineralization and repair processes. However, studies on the effect of Sweroside on osteoblast proliferation and differentiation have mainly focused on cell experiments. *In vivo* models are better able to reflect the mechanism of action of drugs in the whole organism. More *in vivo* studies are needed in the future to further confirm its potential in the treatment of osteoporosis.

In cancer therapeutics, Sweroside has been shown to induce apoptosis in cancer cells by blocking the cell cycle, upregulating pro-apoptotic Bax expression, and downregulating anti-apoptotic Bcl-2 expression. Its ability to target these molecular pathways underscores its potential for integration into anticancer strategies. Furthermore, Sweroside exhibits remarkable efficacy in addressing diabetic nephropathy. By suppressing Pck1 expression, promoting Akt and Erk1/2 phosphorylation, and activating SIRT1-mediated p65 NF-κB deacetylation, it effectively mitigates the progression of this complex disease. This regulatory activity extends to the Akt/BAD signaling pathway, which further contributes to its protective effects on renal function. Importantly, Sweroside has shown significant therapeutic potential in alleviating myocardial injury. And it attenuates inflammatory responses, improves cardiac function, and reduces oxidative stress by targeting the NF-κB/NLRP3 pathway and modulating the Keap-1/Nrf2/ROS axis. Additionally, Sweroside decreases the release of key biomarkers such as creatine kinase-MB (CK-MB) and lactate dehydrogenase (LDH), while suppressing the production of pro-inflammatory cytokines. These effects collectively underscore its capacity to address cardiac damage and enhance recovery following myocardial injury.

In summary, Sweroside’s ability to simultaneously act on multiple targets and pathways highlights its potential as a versatile agent for the treatment of complex diseases. Its broad mechanism of action offers a solid foundation for drug development, paving the way for innovative treatments in areas such as liver disease, osteoporosis, cancer, diabetic nephropathy, and myocardial injury. However, before it is truly applied in clinical practice, the research on Swevoside still needs to be expanded and deepened. Sweroside’s specific mechanisms of action remain incompletely elucidated, the underlying mechanisms governing lipid metabolism regulation, inflammatory response modulation, and antioxidant capacity require further investigation. Current research primarily relies on animal models and *in vitro* experiments. While short-term treatment has shown promising outcomes, the long-term efficacy and potential side effects of Sweroside remain unclear. Future studies are warranted to further explore its mechanisms of action, optimize the drug delivery system, and evaluate its long-term efficacy and safety.

### 7.2 Overcoming pharmacokinetic challenges: bioavailability and toxicity assessment of sweroside

Sweroside has garnered significant attention for its therapeutic potential, yet its bioavailability and toxicity remain critical areas of investigation. *In vivo* studies using animal models have provided valuable insights into its pharmacokinetics, although challenges such as low oral bioavailability persist. Current data indicate that Sweroside’s poor oral bioavailability necessitates the exploration of innovative drug delivery strategies to enhance its therapeutic efficacy. Key approaches include the application of nanotechnology (Huang et al., 2021), structural modification, absorption promoters, and enzyme inhibitors.

For instance, the oil-in-water emulsion method has been employed to DFNPs loaded with secoiridoid glycosides, utilizing PLGA as a controlled-release polymer and pH-responsive polymers such as ES100 and EL30D-55 (Gao et al., 2021). This nanotechnology significantly reduces premature drug release in the stomach and small intestine, achieving controlled release in the colon and prolonging drug residence time. By optimizing polymer ratios, this method not only addresses the limitations of traditional oral formulations but also minimizes systemic absorption and associated side effects, thereby enhancing bioavailability. Such advancements hold particular promise for treating diseases like ulcerative colitis. Structural modifications further augment Sweroside’s pharmacokinetic profile. Esterification, such as the introduction of acetate groups, enhances water solubility and stability, while etherification, including benzyl ether or triphenylmethyl ether modifications, protects hydroxyl groups, improving both stability and lipophilicity (Kong et al., 2022). Cyclodextrin inclusion complexes also enhance water solubility and stability, with β-cyclodextrin increasing compound stability by approximately twofold.

Alternative drug delivery routes, such as intravenous injection or transdermal systems, offer additional solutions to bypass the first-pass effect and improve bioavailability. Transdermal systems, including liposome- or nanocarrier-enhanced plasters, increase skin permeability and enable localized drug delivery. Studies have also explored lipid nanoparticles and hydrogel formulations to improve the penetration of secoiridoid glycosides in the skin, offering potential applications in both medicine and cosmetics (Dąbrowska and Nowak, 2021). Given Sweroside’s anti-inflammatory and anti-melanin properties, its potential in developing multifunctional cosmetics for skin repair, whitening, and inflammation reduction warrants further exploration. Additional strategies, such as the use of surfactants (e.g., Tween 80) to enhance solubility and intestinal permeability, or P-glycoprotein inhibitors (e.g., cyclosporine) to reduce intestinal and hepatic metabolism, further expand the scope for optimizing Sweroside’s bioavailability (Wang M. et al., 2024). Collectively, innovations in formulation technologies, including nanocarriers, controlled-release systems, and local delivery methods, not only address pharmacokinetic limitations but also broaden Sweroside’s applicability across therapeutic and cosmetic fields.

When evaluating the clinical potential of natural products such as Sweroside, balancing therapeutic efficacy with safety is paramount. Dose-dependent toxicity remains a critical consideration in drug development, as even naturally derived metabolites can exhibit adverse effects at high doses (Li et al., 2021). Existing studies suggest that Sweroside exhibits low toxicity under most experimental conditions. For example, in studies on Gentiana asclepiadea extracts rich in Sweroside, no mortality or toxicity-related symptoms were observed at doses as high as 1,600 mg/kg, demonstrating excellent safety. Similarly, acute toxicity experiments with Palicourea rigida roots and leaves showed no toxic effects in mice (Alves et al., 2016). In cytotoxicity assays, Sweroside exhibited no significant toxicity at concentrations up to 160 μM, with only mild cytotoxicity observed at 320 μM, where cell viability remained at 77.0% (Wang et al., 2019). Furthermore, Sweroside isolated from Alsophila stems demonstrated no cytotoxicity at effective doses of 5 μM and 10 µM in TREx-HeLa-Vpr cell experiments (Win et al., 2019).

However, certain studies indicate potential toxicity under specific conditions. For instance, in the brine shrimp lethality bioassay, Sweroside exhibited an LD50 value of 34 µg/mL, suggesting moderate toxicity (Kumarasamy et al., 2003). Additionally, predictive modeling using the IRFMN/ISSCAN-CGX system estimated a carcinogenic risk value of 0.802, highlighting potential long-term risks (Sobot et al., 2020). These findings underscore the need for further validation and comprehensive toxicity assessments. Future research should prioritize long-term toxicity studies across diverse animal models, evaluate the impact of varying formulations and administration routes on toxicity, and investigate underlying mechanisms of toxicity. Moreover, systematic assessments of carcinogenicity and environmental toxicity are essential to ensure the metabolite’s safe application.

In short, while current evidence supports the safety of Sweroside at conventional doses, its toxicity profile and potential risks require further elucidation through rigorous research. Advances in formulation technologies and delivery systems have the potential to overcome existing pharmacokinetic barriers, unlocking new applications for Sweroside in both medicinal and non-medicinal fields. These efforts will lay a solid foundation for the clinical development of Sweroside, ensuring its safety and efficacy in addressing complex diseases and expanding its utility in innovative therapeutic and cosmetic products.

### 7.3 Future research directions and emerging applications of sweroside

Sweroside represents a promising metabolite for therapeutic innovation, with emerging research directions highlighting its potential applications across diverse disease domains. Advances in molecular biology and technology (Li et al., 2023) have provided critical insights into its mechanisms of action, offering new avenues for exploration. For instance, the Caspase family, pivotal mediators of programmed cell apoptosis and inflammation, has been identified as a key target for treating conditions such as neurodegenerative diseases, metabolic disorders, and cancers. Existing evidence suggests that Sweroside inhibits Caspase-1 while promoting the activities of Caspase-3 and Caspase-9, potentially contributing to its therapeutic effects. Additionally, the inhibition of Caspase-2 has been proposed as a targeted strategy to prevent or treat stress-induced fatty liver disease (Kim et al., 2018). This suggests that Sweroside’s capacity to ameliorate obesity and fatty liver may be linked to its regulation of lipid metabolism and inflammatory responses via Caspase-2 inhibition. However, whether its apoptotic effects involve Caspase-6 (Zheng et al., 2020) or other Caspase family members warrants further investigation.

The role of microglial NF-κB overactivation as a driving factor in neuroinflammation and neurodegenerative conditions such as Alzheimer’s disease (AD), amyotrophic lateral sclerosis (ALS), and hypoxic-ischemic encephalopathy (HIE). Suppressing NF-κB activity has emerged as a promising therapeutic strategy to mitigate neuroinflammation and improve clinical outcomes (Anilkumar and Wright-Jin, 2024). Given Sweroside’s potent anti-inflammatory and antioxidant properties, its neuroprotective potential, particularly through modulation of the NF-κB pathway, represents an important direction for future research. This investigation could provide valuable insights into its role in alleviating neurodegenerative pathologies.

In the realm of metabolic diseases, the recent reclassification of non-alcoholic fatty liver disease (NAFLD) to metabolic-associated fatty liver disease (MAFLD) reflects a more comprehensive understanding of its core pathological features (Rong et al., 2023). Unlike NAFLD, MAFLD emphasizes the interplay between hepatic fat deposition and systemic metabolic disorders such as type 2 diabetes, obesity, and metabolic syndrome, which exacerbate the risk of liver fibrosis and cardiovascular complications (Gofton et al., 2023). Current therapies, including GLP-1 receptor agonists and SGLT2 inhibitors, have demonstrated efficacy in managing MAFLD. Sweroside holds significant promise in addressing both NAFLD and MAFLD by enhancing insulin sensitivity, regulating lipid and glucose metabolism, and mitigating inflammation and oxidative stress. Notably, its ability to improve insulin sensitivity may complement the metabolic benefits of GLP-1 receptor agonists, while its antioxidant and anti-inflammatory effects could reduce oxidative stress and inflammation associated with these treatments. Future studies should explore the synergistic effects of combining Sweroside with GLP-1 receptor agonists or SGLT2 inhibitors, particularly in the context of weight management, metabolic regulation, and clinical applications.

Sweroside’s unique anti-inflammatory properties also position it as a candidate for addressing infectious diseases. Recent findings utilizing network pharmacology and molecular docking techniques reveal that Sweroside can bind to key molecular targets, including the main protease (5R82) and 3CL protease (6M2N) of SARS-CoV-2, as well as the androgen receptor (AR, 1T65). These interactions suggest its potential utility in the treatment of COVID-19 and other respiratory diseases (Wang W. X. et al., 2024). By leveraging its anti-inflammatory mechanisms, Sweroside may offer novel therapeutic strategies for managing respiratory infections. Further research should focus on elucidating its molecular targets and pathways to expand its application in infectious and respiratory diseases.

**FIGURE 7:**
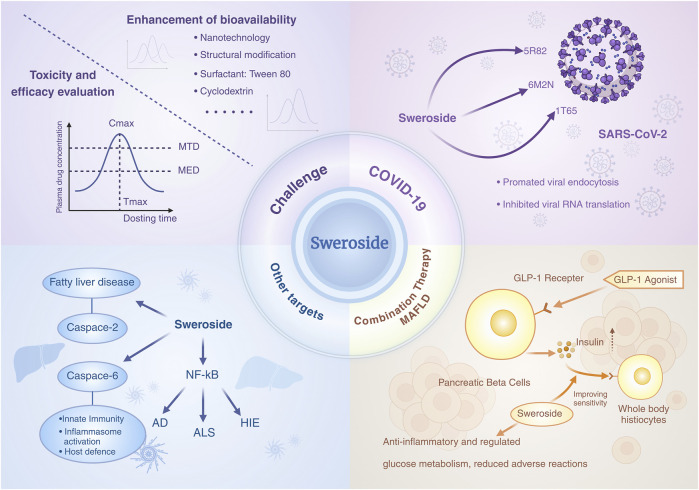
Challenges and future directions for Sweroside. Enhancing Sweroside’s bioavailability, along with rigorous toxicity and efficacy evaluations, remain critical challenges. Further exploration is needed to elucidate its mechanisms in specific diseases and to investigate its potential in combination therapies with other pharmacological agents.

In conclusion, Sweroside exhibits broad therapeutic potential across metabolic, neurological, and infectious disease domains. By integrating modern molecular research with advanced drug development technologies, future studies can deepen our understanding of its mechanisms of action and synergistic effects. These efforts will be instrumental in translating Sweroside’s preclinical promise into clinical applications, paving the way for precision medicine and the development of innovative therapeutics (Figure 7).

## Glossary

n-Butanol: n-BuOH
LPS: Lipopolysaccharide
ALI: Acute Lung Injury
SIRT1: Sirtuin 1
NF-κB: Nuclear Factor Kappa-Light-Chain-Enhancer of Activated B Cells
IκBα: Inhibitor of NF-κB Alpha
NLRP3: NOD-Like Receptor Family Pyrin Domain Containing Protein 3
TNF-α: Tumor Necrosis Factor-Alpha
IL-1β: Interleukin-1 Beta
iNOS: Inducible Nitric Oxide Synthase
COX-2: Cyclooxygenase-2
COX-1: Cyclooxygenase-1
IL-6: Interleukin-6
IKKα: IκB Kinase α
RUNX2: Runt-Related Transcription Factor 2
Cbfa1: Core-Binding Factor Alpha 1
COL1A1: Collagen Type I Alpha 1 Chain
OCN: Osteocalcin
OPG: Osteoprotegerin
PI3K: Phosphatidylinositol 3-Kinase
Akt: Protein Kinase B
p-Akt: Phosphorylated Protein Kinase B
Erk1/2: Extracellular Signal-Regulated Kinase 1/2
GSK-3β: Glycogen Synthase Kinase-3 Beta
Bcl-2: B-Cell Lymphoma/Leukemia-2
Bax: BCL2-Associated X Protein
mTORC1: Mammalian Target of Rapamycin Complex 1
p-S6: Phosphorylated Ribosomal Protein S6
NO: Nitric Oxide
PGE2: Prostaglandin E2
MMP: Matrix Metalloproteinase
ADAMT5: A Disintegrin and Metalloproteinase with Thrombospondin Motifs 5
BCRP: Breast Cancer Resistance Protein
CCl4: Carbon Tetrachloride
5-LOX: 5-Lipoxygenase
BChE: Butyrylcholinesterase
CUPRAC: Cupric Reducing Antioxidant Capacity
FRAP: Ferric Reducing Antioxidant Power
4-NQO: 4-Nitroquinoline 1-Oxide
ROS: Reactive Oxygen Species
MDA: Malondialdehyde
SOD: Superoxide Dismutase
GSH: Glutathione
GSH-Px: Glutathione Peroxidase
HO-1: Heme Oxygenase-1
Keap1-Nrf2: Kelch-Like ECH-Associated Protein 1-Nuclear Factor Erythroid 2-Related Factor 2
CAT: Catalase
D-Gal: ND-Galactosamine
NASH: Non-Alcoholic Steatohepatitis
NAFLD: Nonalcoholic Fatty Liver Disease
MCD: Methionine-Choline-Deficient
AST: Aspartate Aminotransferase
ALT: Alanine Aminotransferase
PPAR-α: Peroxisome Proliferator-Activated Receptor α
CYP7A1: Cytochrome P450 Family 7 Subfamily A Member 1
CYP8B1: Cytochrome P450 Family 8 Subfamily B Member 1
HSD3B7: Hydroxy-Delta-5-Steroid Dehydrogenase, 3 Beta-And Steroid Delta-Isomerase 7
Mrp2: Multidrug Resistance-Associated Protein 2
OSTβ: Organic Solute Transporter Beta
Mdr1: Multidrug Resistance Protein 1
NTCP: Na/Taurocholate Cotransporting Polypeptide
Oatp1a1: Organic Anion Transporting Polypeptide 1A1
Oatp1b2: Organic Anion Transporting Polypeptide 1B2
ANIT: Alpha-Naphthyl Isothiocyanate
ALP: Alkaline Phosphatase
TBIL: Total Bilirubin
DBIL: Direct Bilirubin
TBA: Total Bile Acids
CYP450: Cytochrome P450
CYP3A4: Cytochrome P450 3A4
AC: Adenylate Cyclase
IC50: Half-Maximal Inhibitory Concentration
OVX: Ovariectomy
BMSCs: Bone Marrow Mesenchymal Stem Cells
BMP2: Bone Morphogenetic Protein 2
Pck1: Phosphoenolpyruvate Carboxykinase 1
AML: Acute Myeloid Leukemia
CDK4: Cyclin-Dependent Kinase 4
CDC2: Cell Division Cycle 2
PARP: Poly(ADP-Ribose) Polymerase
JNK/p38 MAPK: c-Jun N-Terminal Kinase/p38 Mitogen-Activated Protein Kinase
DN: Diabetic Nephropathy
HIF1α: Hypoxia-Inducible Factor-1α
BAD: Bcl-2-Associated Death Promoter
ASC: Apoptosis-Associated Speck-Like Protein Containing a CARD
CaMKIIδ: Calcium/Calmodulin-Dependent Protein Kinase II Delta
TAC: Transverse Aortic Constriction
Ang II: Angiotensin II
NGF: Nerve Growth Factor
AchE: Acetylcholinesterase
GPX: Glutathione Peroxidase
AD: Alzheimer’s Disease
TRP-1: Tyrosinase-Related Protein 1
TRP-2: Tyrosinase-Related Protein 2
ERK: Extracellular Signal-Regulated Kinase
BBB: Blood-Brain Barrier
CNS: Central Nervous System
AUC: Area Under the Plasma Concentration-Time Curve
t1/2: Terminal Elimination Half-Life
fe-U: Fraction of Dose Excreted Unchanged in Urine
fu: Fraction Unbound
FOXO1: Forkhead Box Protein O1
Keap-1: Kelch-Like ECH-Associated Protein 1
CK-MB: Creatine Kinase MB
LDH: Lactate Dehydrogenase
PLGA: Poly(Lactic-Co-Glycolic Acid)
DFNPs: Dendritic Fibrous Nano-particles
ALS: Amyotrophic Lateral Sclerosis
HIE: Hypoxic-Ischemic Encephalopathy
MAFLD: Metabolic Associated Fatty Liver Disease
GLP-1: Glucagon-Like Peptide-1
SGLT2: Sodium-Glucose Cotransporter 2
